# (Almost) all of entity resolution

**DOI:** 10.1126/sciadv.abi8021

**Published:** 2022-03-25

**Authors:** Olivier Binette, Rebecca C. Steorts

**Affiliations:** 1 Department of Statistical Science, Duke University, Durham, NC, USA.; 2 Department of Statistical Science, Computer Science, Biostatistics and Bioinformatics, the Rhodes Information Initiative at Duke (iiD) and the Social Science Research Institute (SSRI), Duke University, Durham, NC, USA.; 3 Principal Mathematical Statistician, United States Census Bureau, Washington, DC, USA.

## Abstract

Whether the goal is to estimate the number of people that live in a congressional district, to estimate the number of individuals that have died in an armed conflict, or to disambiguate individual authors using bibliographic data, all these applications have a common theme—integrating information from multiple sources. Before such questions can be answered, databases must be cleaned and integrated in a systematic and accurate way, commonly known as structured entity resolution (record linkage or deduplication). Here, we review motivational applications and seminal papers that have led to the growth of this area. We review modern probabilistic and Bayesian methods in statistics, computer science, machine learning, database management, economics, political science, and other disciplines that are used throughout industry and academia in applications such as human rights, official statistics, medicine, and citation networks, among others. Last, we discuss current research topics of practical importance.

## Teaser

This article reviews entity resolution or record linkage in both statistics and computer science.

## INTRODUCTION

As commonly known in computer science and statistics, entity resolution is the process of taking large noisy databases and removing duplicate entities (often in the absence of a unique identifier) (*1*–*8*). This task has become increasingly more important in science given that the entity resolution task is critical to have more reliable analyses. Since then, entity resolution has been widely studied in many research fields such as statistics, computer science, machine learning (*5*, *6*, *8*–*12*), political and social science (*13*, *14*), medicine and epidemiology (*15*–*20*), official statistics (*21*–*26*), human rights statistics (*27*–*32*), author name disambiguation (*33*–*37*), and forensic science (*38*, *39*), among many other disciplines.

This review is motivated by the long history of entity resolution, its active development in the scientific literature over the years, and its growing relevance throughout many scientific domains. We aim to enable the broader community to understand entity resolution methodology, from its foundation to recent modern developments. Specifically, we focus on social science and official statistics applications of entity resolution such as the U.S. decennial census, casualty estimation in armed conflicts, voter registration data, and the analysis of coauthorship networks. These applications often require uncertainty quantification—rigorous statements of confidence regarding results—as well as principled and interpretable methods that can be subjected to scientific scrutiny. In contrast with other recent reviews of entity resolution, we therefore emphasize probabilistic record linkage as well as recent progress with Bayesian approaches, graphical modeling, and microclustering. Furthermore, given the breadth of the field, we attempt to explain differences between these communities in a meaningful way to bridge this gap. There has been an abundance of contributions due to the database, machine learning, and computer science communities [see surveys (*1*–*8*)]. In contrast to statistical approaches, their focus has typically been on rule-based approaches (*40*, *41*), supervised learning approaches (*42*), hybrid human-machine approaches (*43*, *44*), and scalability (*45*).

Our review is structured as follows. The “Overview of entity resolution” section provides an overview of entity resolution, including impactful applications, terminology and definitions, challenges, and differences among entity resolution disciplines. The “Deterministic record linkage” section discusses rule- and similarity-based approaches, which are popular due to their interpretability and scalability. The “Probabilistic record linkage” section introduces probabilistic record linkage methods that have led to many advancements and extensions. The “Modern probabilistic record linkage” section reviews advancements of modern probabilistic record linkage, which include extensions to the Fellegi and Sunter framework, Bayesian extensions, and semisupervised and fully supervised record linkage methods. Moreover, this is where the most contributions from the machine learning and computer science communities have recently evolved, which contrasts that of the statistical literature. The “Entity resolution as a clustering problem” section reviews clustering-based approaches to entity resolution. We cover clustering tasks that are specifically postprocessing steps, graphical entity resolution methods, and microclustering models. Discussion discusses open research problems, and Supplementary Text provides references to open source software and datasets.

Last, while we attempt to cover almost all of the entity resolution literature, its breadth makes it impossible to cover all of it. Our focus is solely on structured entity resolution, and our focus is not the other subtasks of the data cleaning pipeline (*5*, *6*, *8*). We focus on structured databases as these are more common in statistical applications, where a major goal is ascertaining uncertainty propagation of the structured task, which can be more difficult for unstructured databases. In addition, we seek to strike a balance between many diverse communities working on the same problem that approach the entity resolution task differently. We hope that this article will help build further understanding between the communities and bring them closer together.

## OVERVIEW OF ENTITY RESOLUTION

One of the earliest references to record linkage (here considered synonymous to entity resolution) is from Dunn (*46*), who defined record linkage as the process of assembling pieces of information that refer to the same individual. In 1946, Dunn wrote the following:

“Each person in the world creates a Book of Life. This Book starts with birth and ends with death. Its pages are made up of the records of the principal events in life. Record linkage is the name given to the process of assembling the pages of this Book into a volume.”

In short, record linkage (or entity resolution) seeks to bring together all relevant information about a person, business, or entity. This problem has gathered interest from the scientific community, including in statistics, computer science, machine learning, database management, finance, fraud detection, political science, official statistics, and medicine, among many others. In this section, we provide an overview of some of the most important applications of entity resolution that have been used throughout science in recent years for structured data (“Impactful entity resolution applications” section). In addition, we review terminology in entity resolution and the data cleaning pipeline (“Terminology and definitions” section), as well as challenges in the entity resolution task (“Challenges of entity resolution” section). Last, we review critical differences between disciplines regarding how researchers approach the entity resolution task.

### Impactful entity resolution applications

We now review applications across science that have motivated major developments in entity resolution.

#### Decennial census

One important and timely topic is one that faces the U.S. Census Bureau each decade when they attempt to count all the individuals in the population. This enumeration is used to allocate resources for roads, schools, projects, and apportion representation of legislators. Unfortunately, it has been shown difficult to accurately enumerate such a population using an optional census, and response rates are often quite low. Furthermore, some individual may be counted multiple times. For example, an individual that owns three houses might accidentally fill out three census forms. As another example, individuals in group quarters (such as universities and prisons) are often double counted by their “group” and a family member/parent/guardian (*47*). Deduplication is thus needed to obtain an accurate enumeration, with new methodology from the machine learning and statistical literature being recently proposed to this end (*48*). This methodology is scalable while providing exact error propagation throughout the blocking and the entity resolution task (*48*).

#### Human rights statistics

In this section, we review two case studies in human rights statistics.

##### Documented identifiable deaths in El Salvador

Between 1980 and 1991, the Republic of El Salvador witnessed a civil war. There are three databases available for this conflict, where duplications occur within and across each of the databases. The first two databases were collected during the conflict, whereas the third database was collected after the conflict. The first two databases contain reports on documented identifiable victims. The first source, El Rescate (ER-TL), a nongovernmental organization (based out of Los Angeles, CA), collected electronic data from published reports during the civil war (*49*). The second source, Comission de Derechos Humanos de El Salvador (CDHES), collected testimonials on violations from 1979 to 1991 (*50*). The third source contains reports on documented identifiable victims after the civil war. After the peace agreement in 1992, the United Nations created a Commission on the Truth (UNTC), which invited citizens to report war-related human rights violations. Hence, victims can be duplicated in these datasets.

##### Syrian conflict

One case study that has been of interest is the ongoing Syrian conflict. To our knowledge, the Human Rights Data Analysis Group (HRDAG) provided the first published work in this domain (*28*, *51*, *52*). There are four sources that collected data during the same time period—the Syrian Center for Statistics and Research (CSR-SY), the Syrian Network for Human Rights (SNHR), the Syria Shuhada website (SS), and the Violation Documentation Centre (VDC). Each source provides documented identifiable deaths in the conflict. Attributes available are full Arabic name, gender, death location, and date of death. HRDAG has labeled the dataset, as outlined in their paper (*51*).

Both applications have proven to be extremely important to the development of the entity resolution literature. The El Salvadoran application is important as it is an application to small-scale human rights data, where some attributes contain a great deal of noise due to the way the information was collected. Given uncertainty in the data, it is natural in this application to use fully unsupervised approaches. Furthermore, uncertainty propagation of the entity resolution process has been demonstrated to be important in recently published case studies (*30*–*32*, *50*, *53*). In addition, the Syrian dataset is also important given that researchers have proposed a near-linear time algorithm for unique entity estimation that provides uncertainty quantification of the number of documented identifiable deaths (*54*).

#### Estimation of voters in North Carolina

Another application that has been recently used in science has been voter registration databases, which are publicly available (and often online) in the United States. For example, the North Carolina State Board of Elections (NCSBE) publicly posts its voter registration database (www.ncsbe.gov/). This dataset contains rich information, such as first and last name, year of birth, phone number, and address. However, the voter registration number is often duplicated due to people moving, getting married, and various other reasons. See Table 1 for examples of public records from this dataset. The process through which voter registration records are matched with other official records can have a profound influence on one’s ability to vote. Georgia’s controversial “exact match” law (*55*), which was slightly changed in 2019, required an exact match between voter registration records and records from the Department of Driver Services or the Social Security Administration to validate voter registrations. For instance, typographical errors, different spellings of the same name, or outdated records could place a voter registration on hold. In 2017, about 670,000 registrations were canceled as a result (*56*). Enamorado (*57*) showed how this law could predominantly affect non-white voters [see also (*58*)]. This application illustrates the need for uncertainty quantification and fairness analyses.

**Table 1.** Example of public NCSBE records retrieved from for the county of Durham in North Carolina and corresponding to unique voter registration numbers. **www.ncsbe.gov** Some fields have been omitted for brevity, including ZIP code, phone number, and voter registration number. Street addresses have been permuted with other individuals to preserve some anonymity.

**Name**	**Street address**	**Age**	**Sex**	**Race**	**Birth**	**Party**
Domineck Q. AAshad Jr.	914 Monmouth Ave #3	26	M	B	–	LIB
Domineck Q. AAshad Sr.	1408 Auburndale Dr	55	M	B	NY	DEM
Xiomara A. Martinez	1715 Cole Mill Rd	31	F	O	HL	REP
Xiomara A. Martinez	2923 Forrestal Dr	31	F	O	HL	–
Virginia L. Mullinix	749 Ninth St #480	101	F	W	PA	REP
Jacqueline D. Fuller	141 Bagby LN	54	–	–	–	DEM
Jacqueline Fuller	905 Cook Rd	56	F	B	NC	DEM

Remark: While our motivating examples have highlighted individuals, we would like to note that entity resolution can apply to businesses and objects.

#### Inventor and author disambiguation

Author disambiguation is the problem of identifying documents that have been written by the same author. Disambiguated data are informative for the study of innovation and productivity (*59*). Many datasets have been used broadly throughout statistics, computer science, and machine learning to develop algorithms for this task. These are fully labeled (or partially labeled) benchmark datasets that tend to illustrate success on new methods. On the other hand, there are research questions of interest that can be posed from fully unsupervised author disambiguation using such datasets that do not contain any training labels, where the labels would also need to be provided in a principled manner that would not be biased toward any proposed algorithm.

Section S3 reviews recent benchmark datasets, where one can find a more comprehensive review of author disambiguation datasets and methodology in (*60*). Section S3 reviews research datasets, which typically do not have unique identifiers or went through an intensive and well-documented manual labeling process. In general, there does not appear to be a strong consensus within the author disambiguation community regarding a standard on benchmark datasets, which holds true for the rest of the entity resolution literature. In addition, most of the entity resolution datasets (including the hand labeled pairs) are not easily reproducible by authors, which presents a strong need for communities to set forth more clear standards regarding how such datasets should be created such that datasets do not favor one proposed method over another.

These applications have proven to be impactful as researchers and industry leaders have been able to consider many types of methods, such as deep learning models as well as semisupervised and fully supervised methods, among others, illustrating the strength of these approaches for this particular application.

### Terminology and definitions

In this section, we introduce terminology that will be used throughout the article that is commonly known and used in the existing literature (*5*, *6*, *61*). In addition, we formally define the entity resolution problem.

A database (file) is a collection of records. A record in a database contains attributes (fields or features), such as given name, family name, date of death, and municipality. Here, we assume that each record refers to an entity (person, object, or event) with respect to which we want to aggregate relevant information. Entity resolution is the problem of identifying records that refer to the same entity, such as identifying individual victims among recorded deaths that contain duplication. Two records that refer to the same entity are said to be coreferent (a link) or to be non-coreferent otherwise (nonlink). Entity resolution can be framed as clustering records according to the entity to which they refer or, equivalently, of identifying coreferent record pairs. This is also referred to as record linkage, deduplication, data matching, instance matching, and data linkage.

Remark: Performing entity resolution on a single database that contains coreferent records (duplicates) is often referred to as deduplication (or duplicate detection). This is the simplest case, where any structure corresponding to the source of each record is ignored. When the data are part of two databases, with duplication across but not within databases, the problem is referred to as bipartite record linkage.

#### The data cleaning pipeline

Entity resolution is usually thought of one stage in the data cleaning pipeline (*2*, *5*, *61*) represented below

attribute alignment→blocking→entity resolution→canonicalization (1)

In the first stage, attribute or schema alignment, records are parsed to identify a set of common attributes among the datasets. In the second stage, blocking, similar records are grouped into blocks. Only records appearing in the same block will then be compared; records that do not appear in the same block are automatically determined to be nonmatches. In the entity resolution stage, coreferent records are identified. Last, in the fourth stage, merging, data fusion, or canonicalization, entities resolved as matches in the third stage are merged to produce a single representative record. The focus of this review article is on structured entity resolution and not on the other stages of the pipeline. For surveys of the entire pipeline, we refer to (*5*, *6*, *8*).

### Challenges of entity resolution

Entity resolution tasks face a trade-off between (i) scaling to large databases, (ii) providing uncertainty propagation of the entity resolution task through all stages of the data cleaning pipeline, and (iii) proposing methods that account for the distortions and errors found in the databases. For databases with a combined total of *N* records, there are *N*(*N* − 1)/2 pairs of records that must be considered as being possibly coreferent. Evaluating each pair is therefore not scalable as the number of records grows. Most entity resolution methods avoid comparing all pairs by blocking. While this increases the computational speed, uncertainty cannot be propagated exactly from the blocking stage to the entity resolution stage, as shown in Eq. 1. Specifically, the entity resolution task will inherit any errors from the blocking task, some of which cannot be resolved. On the other hand, one can achieve exact uncertainty propagation by building a joint blocking and entity resolution model; however, this typically results in slower computational run times. Last, when dealing with data that contain distortions, typographical errors, and noise, generative methods have seen success. Such models are typically more complex and thus typically may not scale to large databases. The trade-off between (i), (ii), and (iii) must be evaluated by the user based on each motivating application so that the most appropriate method can be chosen.

### Differences in entity resolution disciplines

As previously stated, entity resolution is a broad interdisciplinary field of research. Given our motivating applications, our review paper is specifically concerned with structured entity resolution problems, where records are composed of clear attributes such as names and phone numbers. This can be contrasted with unstructured entity resolution, where entity instances may contain textual descriptions or images. In addition, we focus on entity resolution approaches that provide uncertainty quantification, such as probabilistic record linkage, Bayesian approaches, graphical modeling approaches, microclustering models, and semi- and fully supervised approaches, among others. These methods are needed in scientific applications where all sources of uncertainty that may affect the validity of results must be accounted for. The main challenge, in this case, is to properly quantify this uncertainty and to account for it in downstream analyses. This is a very different focus than what is found in most of the computer science, machine learning, and database management literature, where the main goal is to address big data challenges such as large amounts of data, continuously evolving databases, and streaming data. Without losing sight of our motivating applications, we have attempted to highlight approaches from both disciplines to bring these communities (and others) closer together.

## DETERMINISTIC RECORD LINKAGE

In practice, the most commonly used entity resolution methods are based on a series of deterministic rules involving the comparison of record attributes. These are called deterministic, rule-based, and similarity-based approaches. A simple example is exact matching, where two record pairs are linked if they agree on all common attributes. This strict matching condition can be relaxed by allowing mismatch on a fixed number of attributes, by using disjunctions of exact matching rules, and by using similarity functions. These rules can also be learned from data, bringing us closer to the topic of probabilistic record linkage discussed in the “A theory of record linkage” and “Modern probabilistic record linkage” sections. However, the differentiating characteristic of deterministic approaches is that they do not account for uncertainty in the matching process. No probability model is used and no level of confidence is provided for the matching status of record pairs.

Record attributes are often distorted by noise (due to data entry errors, variant spellings, outdated records, etc.). Naturally, linkage rules should account for slight differences between attributes. One simple way to quantify such differences for names, addresses, and other textual attributes is via string distance functions. For westernized words, edit distances such as the Levenshtein distance (*62*, *63*) are used to account for deletions, insertions, and substitutions. The Jaro-Winkler distance (*21*, *64*) works well for the comparison of short strings such as name. In addition to edit distances and their variants, token-based similarity measures such as the Jaccard similarity and the cosine similarity are often used when dealing with unstructured text and longer strings. We refer the reader to (*65*, *66*) for more information regarding simple string distance functions.

In practice, rule-based systems are carefully crafted for the application at hand. As noted in (*67*, *68*), a large body of work in the computer science and database communities is devoted to rule-based methods. This includes the specification and learning of linkage rules (including learning string similarity functions) (*40*, *69*–*74*), the use and efficient computation of similarity functions (*75*–*77*), the use of indexing structures and algorithms for efficient execution of entity resolution in large databases (including blocking and filtering) (*78*–*81*), the use of clustering techniques to resolve linkage transitivity (see the “Clustering as a post-processing step” section) (*82*–*84*), and the integration of matching records (data fusion) (*61*, *85*). Notably, Benjelloun *et al*. (*86*) provide algorithms to minimize the number of pairwise comparisons when matching and merging records. This literature is thoroughly reviewed as part of (*1*, *6*–*8*). These methods allow the use of entity resolution at much larger scales than what is possible using probabilistic approaches of the kind discussed in the following sections. In addition, they can be used as part of a blocking stage to scale up other entity resolution methods that account for uncertainty.

While deterministic approaches are appealing for their simplicity, interpretability, and computational scalability, empirical studies comparing deterministic and probabilistic record linkage techniques used for epidemiological research have shown consistent improvements of probabilistic methods over deterministic approaches (*87*–*91*). Other literature has surveyed probabilistic versus deterministic methods more broadly in terms of comparisons, also finding improved performance with probabilistic methods (*92*, *93*). While being application specific, these evaluations showcase the potential of probabilistic approaches when linking noisy data, both accounting for uncertainty and providing good performance.

## PROBABILISTIC RECORD LINKAGE

We now take a step back and turn to the earliest published works on probabilistic record linkage. These works have laid down foundations for the field, which we use as the basis of our discussion of modern methodology. First, we discuss the work of H. Dunn who defined record linkage, leading to the first algorithm solution by Newcombe *et al.* (*94*) (‘Dunn’s “Book of Life” and early references’). Next, we discuss in depth the Fellegi-Sunter record linkage framework (*95*) (“A theory of record linkage” section). This framework provides a principled statistical model for record linkage that is still widely used today. It has the notable property of requiring no training data for record linkage—it is entirely unsupervised. Since its introduction in 1969, the Fellegi-Sunter framework has been widely studied, extended, and even recently reinvented (*96*). These extensions, such as more flexible modeling, Bayesian propagation of uncertainty, and semisupervised learning, are discussed afterward in the “Modern probabilistic record linkage” section.

### Dunn’s “Book of Life” and early references

As previously mentioned, the concept of record linkage (here used as a synonym to entity resolution) first appeared in a paper by Dunn (*46*) in 1946. In the context of administering governmental programs and services, he defined record linkage as the process of assembling pieces of information that refer to the same individual.

Interestingly, Dunn framed record linkage as an entirely logistical problem: If birth certificate numbers were widely used, then any centralized index would effectively bind individual records into this “book of life.” However, the reality is that birth certificate numbers, or other unique identifiers for that matter, are not widely used. Records collected from different organizations, at different times, and for different purposes usually cannot be trivially matched together. Record linkage thus becomes an algorithmic problem—what can best be used to identify records that refer to the same individual, given noisy, uncertain information?

This is a problem that Newcombe *et al.* (*94*) faced in 1959 when trying to match birth and marriage records for demographic studies (*97*–*100*). The authors proposed, to our knowledge, the first automated record linkage method, which did not require a unique identifier. Specifically, they used domain knowledge of last names, first initials, birthplaces, ages (of some records), and location of child birth/marriage events. While no single one of these pieces of information was entirely reliable, together they could be used for accurate record linkage. The idea of their method was quite simple and had two steps. In the first step, the authors used blocking. Specifically, to account for variations in spelling, records were blocked (indexed) on the basis of the Soundex coding of the names. Note that the Soundex coding scheme was introduced by M. K. Odell and R. C. Russell [see U.S. patents 1261167 (1918) and 1435663 (1922)]. It codifies names by the first letter and by a string of three numbers, with the property that phonetically similar names often share the same code. Table 2 provides an example of attribute information from compared marriage and birth records from the original (*94*). Second, when Soundex coding agreed between two records, they computed a likelihood ratio comparing the hypothesis that the record pair were a match to the hypothesis that they were not. If this likelihood ratio exceeded a threshold, then the two records were linked (declared coreferent); otherwise, they were not linked (declared non-coreferent). Studies of the accuracy of the linkage showed that about 98.3% of the true matches were detected and about 0.7% of the linked records were not actual matches. In terms of computational speed, 10 records could be linked every minute on the Datatron 205 computer.

**Table 2.** Example of attribute information from marriage and birth records. This table is adapted from table 1 of (*100*) and translated from French to English. AB and PE represent the Canadian provinces of Alberta and Prince Edward Island. Only the initials of the first and middle names are provided in these data.

**Attribute information**	**Marriage record**	**Birth record**
Husband’s Soundex name code	A300	A300
Wife’s Soundex name code	B600	B600
Husband’s family name	**Ayad**	**Ayot**
Wife’s family name	Barr	Barr
Husband’s initials	J Z	J Z
Wife’s initials	**M** T	**B** T
Husband’s birth province	AB	AB
Wife’s birth province	PE	PE

In short, the work of Newcombe *et al.* (*94*) introduced key ideas for record linkage in an application to demographic data, where blocking (indexing) was used to make the problem computationally tractable. They proposed an informal statistical approach based on a likelihood ratio test, where the pipeline was fully automated and required no training data.

### A theory of record linkage

We now turn to the Fellegi-Sunter framework (*95*) introduced in 1969 and that formalizes the approach of Newcombe *et al.* (*94*) in a decision-theoretic framework. We will define the likelihood ratio of the type used by Newcombe *et al.* and we will see how records can be linked while controlling fixed error rates. Furthermore, we will review the Fellegi-Sunter probability model, its interpretation, and its underlying assumptions.

The decision model of the Fellegi-Sunter framework considers records in independent pairs. For a given pair of records, three possible actions are considered: to link, to possibly link, or to not link. The goal is to minimize the number of possible links while controlling for type I and type II error rates (false match rate and false nonmatch rate). In the Fellegi-Sunter framework, an optimal linkage procedure attains the specified error rates while minimizing the number of possible link assignments. A “fundamental theorem for record linkage” demonstrated by the authors shows that the optimal linkage procedure corresponds to thresholding a likelihood ratio.

The likelihood ratio is defined as follows. Let γ be the comparison vector used to represent the level of agreement/disagreement between two specified records. In practice, γ is usually decomposed as γ = (γ_1_, γ_2_, …, γ*_k_*), where each γ*_i_* corresponds to a comparison between a particular attribute (name, age, etc.) of the record pair. One can consider binary comparisons, where γ*_i_* ∈ {0,1} represents agreement or disagreement between record attributes, as well as more detailed comparisons involving the specific value for which there is an agreement (such as γ*_i_* = “ initials agree and are J & M”). Now, let *m*(γ) be the probability of observing the comparison vector γ for two records that are an actual match, and let *u*(γ) be the probability of observing the comparison vector γ for two records that are not a match. The likelihood ratio is then defined as *m*(γ)/*u*(γ), and its logarithm *W*(γ) = log (*m*(γ)) − log (*u*(γ)) is called the matching weight.

Two unsupervised methods are proposed by Fellegi and Sunter to estimate the *m* and *u* probabilities. In both methods presented below, the authors assumed conditional independence between the attribute comparisons $\{\gamma_{i}\}{}_{i=1}^{k}$ given the true underlying match/nonmatch status of the record pairs.

First, the authors considered detailed comparison vectors, which provide both an indication of agreement or disagreement for each attribute and a precise shared value in the case of an agreement. This allows one to exploit specific information about the record’s attributes. For instance, two records agreeing on the less common name “Xander” are more likely to be a match than two records that only agree on the first name “John.” In applications, it is often helpful to exploit such frequency information. Under this assumption, the authors used the frequency distribution of the record’s attributes, together with prior information about error rates, to obtain estimates of the *m* and *u* probability distributions.

Second, the authors considered binary comparisons, where each γ*_i_* is a binary variable indicating agreement or disagreement with the records’ *i*th attribute. The distributions *m* and *u* can then be estimated from the observed frequencies of agreement or disagreement between these fields. In particular, they derived analytical formulas to estimate *m* and *u* when only three fields are under comparison.

Winkler (*101*) extended the above estimation methods by proposing the use of the expectation-maximization (EM) algorithm to estimate the *m* and *u* distributions both in the context of detailed comparisons between fields (where particular agreement values are also taken into consideration) and binary comparisons. Independently, Jaro (*21*) proposed the EM algorithm for binary comparisons and considered its application for matching the 1985 test census (dress rehearsal) of Tampa, Florida, to an independent post-enumeration survey to evaluate the census coverage.

### Interpretation of the probability model

While the Fellegi-Sunter approach was introduced in a decision-theoretic framework, it can be interpreted more easily through its underlying probability model, where the comparison vectors γ are distributed as the following mixture model

p(γ)=λm(γ)+(1−λ)u(γ)

where λ > 0 is the probability that a randomly chosen comparison vector corresponds to a matching pair of records. The methods proposed by Fellegi-Sunter, as well as the EM algorithm proposed by Jaro (*21*) and Winkler (*101*), provide estimates of the parameters λ, *m*, and *u*.

Denote a true match by *M*. Using the Bayes rule, one can express the probability that two records match given their comparison vector γ, as

$$p(M|\gamma )=\lambda m(\gamma )/p(\gamma )=1-{\left(1+\frac{m(\gamma )}{u(\gamma )}\frac{\lambda }{1-\lambda }\right)}^{-1}$$ (2)

The left-hand side of Eq. 2 is the posterior probability of a match, and the right-hand side shows how it can be obtained as a monotonous transformation of the Fellegi-Sunter likelihood ratio *m*(γ)/*u*(γ). Therefore, as noted in (*102*), thresholding the posterior probability to assign links is equivalent to using a likelihood ratio test and the Fellegi-Sunter optimality result also applies in this context.

### Assumptions of Fellegi-Sunter

The Fellegi-Sunter framework relies on crucial simplifying assumptions. The first assumption is that comparison vectors between the record pairs should be independent from one another. This is usually not satisfied in practice. For example, when Newcombe *et al.* (*94*) linked birth and marriage records, it was known that two different marriages could not result in the same birth. This constraint induces dependencies between comparison vectors, and applying the Fellegi-Sunter procedure can lead to impossible linkage configurations when this is not taken into consideration. Generally, any linkage that does not satisfy transitive closure is impossible—knowing that *a* links to *b* and that *b* links to *c* should entail that *a* also links to *c*.

The second assumption is that the *m* and *u* distributions are known or can be adequately estimated. To be practically feasible, their estimation relies on simplifying assumptions that usually do not hold. For one thing, the estimation methods discussed so far require conditional independence between the comparison of different record attributes, given the true match/nonmatch status of the record pairs. Smith and Newcombe (*103*) first remarked that this conditional independence assumption may not hold in practice. Thibaudeau (*104*) [see also (*105*–*107*)] proposed log-linear models with interaction terms to account for dependencies between field comparisons and showed improved performance in some applications. Given that the assumptions of Fellegi-Sunter are often not satisfied, this has led to many extensions in the literature, which we review in the “Modern probabilistic record linkage” section.

### Modern probabilistic record linkage

In this section, we review modern probabilistic record linkage, which includes extensions to the Fellegi-Sunter framework, Bayesian variants of Fellegi-Sunter, as well as semisupervised and fully supervised classification approaches.

### Extensions of Fellegi-Sunter

In many applications, the procedures neither of Fellegi-Sunter nor of Tepping are used to set classification thresholds. According to Belin and Rubin (*108*), for the matching of the 1990 Census with the post-enumeration survey, thresholds were set “by ‘eyeballing’ lists of pairs of records brought together as candidate matches.” Part of the reason is that the error rates fixed in the Fellegi-Sunter framework, as well as the false match rates estimated using Eq. 2, are not attained in practice (*105*, *106*, *108*, *109*). This is due to the various simplifying assumptions and estimation errors involved in the application of such models. Therefore, methods using training data (classified record pairs) have been proposed to automate and improve the choice of tuning parameters in probabilistic record linkage.

For instance, Belin and Rubin (*108*) proposed to calibrate thresholds and error rates by using training data to fit a mixture model to the matching weight distribution. This allowed the authors to quantify uncertainty about the linkage’s error rates and to calibrate the Fellegi-Sunter thresholds. Nigam *et al.* (*110*) showed how training data could be combined with unlabeled data to improve the estimation of the *m* and *u* distributions using the EM algorithm for text classification. Building on the same semisupervised framework, Winkler (*111*, *112*) and Larsen and Rubin (*102*) considered fitting more complex models, allowing dependencies between field comparisons. Last, Enamorado *et al.* (*14*) have scaled the seminal Fellegi-Sunter model to large databases, where they incorporated auxiliary information into the merge and post-merge analyses (*113*).

### Bayesian Fellegi-Sunter

In the context of entity resolution, Bayesian methods provide a way to quantify and propagate uncertainty for the joint linkage structure of a set of records. Furthermore, Bayesian methods allow the incorporation of prior knowledge, such as linkage transitivity, into analyses. These properties have made them popular in inferential and scientific applications, where uncertainty must be taken into account to reach sound conclusions and where prior knowledge is often available. This section reviews Bayesian record linkage methodology, which extends the Fellegi-Sunter framework.

Fortini *et al.* (*23*) extended the seminal work of Fellegi and Sunter (*95*) in the special case of bipartite record linkage. The authors assumed a prior on the “matching pairs,” a prior on the “matching configuration matrix,” and a Dirichlet prior on the *m* and *u* distributions. Here, the matching configuration matrix, or coreference matrix, indicates the linkage structure between two databases. That is, if we denote by *i* a record in the first database and *j* a record in the second database, then this matrix has entries *c*_*i*, *j*_ ∈ {0,1}, with *c*_*i*, *j*_ = 1 if records *i* and *j* are linked and *c*_*i*, *j*_ = 0 otherwise.

More recently, Sadinle (*30*) proposed the first Bayesian Fellegi-Sunter model, where he assumed two databases and a de-deduplication scenario. He assumed a likelihood similar to that of Fortini *et al*. (*23*). Sadinle (*30*) considered a partitioning approach that allows transitive closures to be satisfied. This allows quantification of uncertainty about the partition of records through a posterior distribution. In later work, Sadinle (*31*) extended the above framework for bipartite record linkage. In addition, the authors derive Bayes estimates under a general class of loss functions, which provides an alternative to the Fellegi-Sunter decision rule. Both the work of Sadinle (*30*, *31*) apply their proposed methodology with deterministic blocking rules to the case study on human rights in El Salvador. In addition to proposing new methodology, Sadinle (*30*) performed hand matching on a small set of the dataset such that pairwise evaluation metrics could be used. The work of Sadinle (*30*, *31*) has been extended by Wortman (*114*), where the author accounts for dependencies between fields and for heterogeneity in the comparison vector distribution.

One difficulty facing Bayesian Fellegi-Sunter is their computational burden. In other recent work, McVeigh *et al*. (*13*) considered this issue by proposing a blocking approach based on simpler probabilistic record linkage techniques. That is, the output of more simple non-Bayesian probabilistic record linkage is used to perform “post hoc blocking,” after which a Bayesian Fellegi-Sunter method is used for coherent modeling and uncertainty quantification. This allows the authors to scale their proposed method to voter registration and census datasets with millions of entries.

### Semi- and fully supervised classification approaches

The approaches of (*108*, *110*–*112*) and (*102*) discussed in the “Extensions of Fellegi-Sunter” section were semisupervised (*115*). Semisupervised methods use a relatively small amount of manually classified record pairs, known as labeled pairs, to improve upon unsupervised probabilistic record linkage. In this section, we review semisupervised methods and fully supervised methods, which focus on classifying record pairs as a first step to entity resolution.

Note that the use of training data in entity resolution can be a complex problem. In many statistical applications, such as with the El Salvadoran dataset, ground truth is not available. The closest available data for use in training might come from one or more human experts through costly review processes. However, despite best efforts, experts can be subject to errors, biases, and uncertainty. Furthermore, the sampling process from which labeled data are obtained is highly influential and must be accounted for. These issues are the topic of broad research on sampling/querying, crowdsourcing, active learning, and performance evaluation for entity resolution. Here, we only briefly touch on these topics, instead focusing on methods that assume a single set of reliable labels.

#### Semisupervised approaches

Following the terminology of Chapelle *et al*. (*115*), we consider three types of semisupervised approaches. First, generative semisupervised approaches target the joint likelihood of the labeled and unlabeled data as in the study of Nigam *et al.* (*110*) and Larsen and Rubin (*102*). Building on this framework, Enamorado (*116*) proposed an active learning algorithm that iteratively requests labels for specific record pairs. Other active learning approaches are proposed in (*117*–*121*). Second, change of representation semisupervised approaches use unsupervised learning as a first step to summarize the data (such as performing dimensionality reduction), before using a supervised algorithm for further analysis. For instance, Belin and Rubin (*108*) used the unsupervised Fellegi-Sunter framework to obtain univariate matching weights for all record pairs, before using labeled examples to fit a mixture model to the matching weights. This allows the authors to calibrate the model and potentially select better thresholds. Third, self-learning algorithms generalize the semisupervised EM algorithm considered in (*110*) and (*102*) to model-free classifiers. In this framework, Kejriwal and Miranker (*122*) combined self-learning and boosting of random forests and multilayer perceptrons to obtain good performances on entity resolution tasks using only small amounts of labeled pairs.

#### Fully supervised approaches

Fully supervised methods do not exploit information provided by unlabeled examples; instead, they rely on larger numbers of labeled pairs. Given the significant class imbalance when considering record pairs (very few pairs match), vast amounts of reliable training data or carefully selected training data are required for the use of these methods. These training data may come from crowdsourcing (*43*, *44*, *117*, *123*, *124*) or from extensive manual record linkage efforts (*125*–*127*), or it may be automatically generated using unsupervised methods to obtain an approximate training set (*128*–*130*). In practice, the amount of reliable training data necessary to train sophisticated learning algorithms such as deep neural networks (*42*, *131*–*136*) is not always available for entity resolution tasks. For instance, Kooli *et al*. (*134*) use more than 10 million examples of labeled record pairs (corresponding to more than 3000 resolved individual records) in an application to train deep neural networks. More recently, Kasai *et al*. (*135*) considered the issue of training deep neural networks for entity resolution with fewer labels, using active and transfer learning. The use of deep learning techniques in entity resolution is especially promising in application to unstructured or textual problems [see (*42*, *137*, *138*)], where, for instance, pretrained language models can be used (*139*). For structured entity resolution, simple classifiers [such as logistic regression, decision trees, random forests, Bayesian additive regression trees, and others (*140*)] are often preferred.

To give an example of how such methods are used in practice, consider the work of Ventura *et al*. (*141*), a case study for inventor disambiguation in the bibliographic database of U.S. Patent and Trademark Office (USPTO) patents. The authors proposed a supervised method based on random forests for deduplication. Their training data were constructed from the curriculum vitae of inventors in the field of optometrics as well as from a previous study on “superstar” academics in the life sciences (*126*, *142*). This allowed them to evaluate the performance of previous methods used in this application (*59*, *143*) and to train their random forest classifier on labeled comparison vectors of record pairs. Afterward, applying their entity resolution approach to other records in the USPTO database consisted of a four-stage pipeline. First, they use blocking where, in each block, they calculated comparison vectors for each record pair. Second, the authors calculated the predicted probability of a match using their random forest classifier applied to these comparison vectors. Third, the predicted probability was converted into an estimate of the dissimilarity between each pair of records. Fourth, the authors used single linkage hierarchical clustering corresponding to the dissimilarity scores in the previous step to enforce transitive closures among record pairs. Clusters were determined by cutting the dendogram (tree) at a threshold. Last, all the clustering results were combined across blocks to obtain a final set of deduplicated records. Such clustering approaches to entity resolution, used either directly or as a follow-up to pairwise classification, are discussed next in the “Entity resolution as a clustering problem” section.

### Active learning

Active learning models are based on semisupervised or fully supervised models. Instead of using a predetermined set of training data, however, they interactively request informative labeled data from an expert labeler. Their goal is to provide a balance between automation and human interaction, although this balance is difficult to quantify. These models perform well on many entity resolution applications—given the large class imbalance in entity resolution tasks, informative training data may be difficult to choose a priori (*144*). The active learning workflow for entity resolution is nicely reviewed in (*8*), where the authors consider early and modern approaches in the literature.

## ENTITY RESOLUTION AS A CLUSTERING PROBLEM

The methods discussed so far focused on estimating the probability of a match between pairs of records given their comparison vector. This pairwise match probability provides a measure of uncertainty about specific links, where the corresponding false match and false nonmatch rates (or precision and recall) are pairwise evaluation metrics of performance. With the exception of Bayesian Fellegi-Sunter, these methods treat record pairs as being independent of one another, without accounting for the consequences of transitivity or other constraints on the linkage structure. This limits their practicality when linking more than two databases (and dealing with applications that have duplication across/within databases). Much of the literature has therefore advocated for a clustering-based approach to entity resolution and deduplication, which can integrate multiple databases (*30*, *31*, *48*, *82*, *145*–*152*). In this context, the goals shift. Instead of linking record to record, the goal is to cluster records to their true (unknown, latent) entity.

A large portion of this literature uses clustering as a second step to probabilistic record linkage to enforce transitivity of the output (*6*, *83*). Other clustering approaches are model-based, and in particular, we focus on graphical entity resolution in the “Graphical entity resolution” section. By probabilistically modeling the relationship of records to the latent entities to which they refer, these methods naturally provide uncertainty quantification regarding the clustering structure (*149*, *153*). Last, entity resolution can be viewed as what we refer to as a microclustering problem, meaning that the size of the latent clusters grows sublinearly as the number of records grows. This means that entity resolution does not experience power law (linear) growth as many traditional clustering tasks. We discuss microclustering in the “The microclustering property” section.

### Clustering as a post-processing step

Many clustering approaches to entity resolution are based on pairwise similarities, pairwise match probabilities, or determined links and nonlinks. Therefore, these can be seen as post-processing the result of other pairwise record linkage procedures. They are used to resolve intransitivites in the linkage method and ensure a coherent output. There is a vast literature on the subject; we only review a selection of the proposed methodology for entity resolution. We refer the reader to (*3*, *5*, *83*, *154*) and (*6*) for more exhaustive reviews.

One of the first references in this area is Monge and Elkan (*82*), who framed entity resolution as a clustering problem. Specifically, they proposed that one should detect the connected components in the undirected graph of pairwise links [see also (*155*, *156*)] using a dynamic connectivity structure. Pairwise links were determined iteratively. At any given step, only records that were not in the same connected component were compared to determine the match/nonmatch status. It allowed the authors to resolve in transitivities in pairwise matching while avoiding superfluous comparisons. The idea of clustering through connected components is computationally efficient and has recently been exploited as part of a blocking stage in the study of McVeigh *et al.* (*13*). A more sophisticated technique, correlation clustering (*157*), maximizes the number of links within clusters plus the number of nonlinks across clusters. This approach was originally introduced in the context of document classification. The number of clusters is determined from an objective function, which is one advantage of this method. However, correlation clustering is NP-hard (*157*, *158*), and in practice, variants and approximate solutions are used (*83*, *159*–*161*). Another approach is hierarchical agglomerative clustering (*140*, *162*), which Ventura *et al.* (*147*) advocated in conjunction with ensemble classifiers for large-scale entity resolution. Ventura *et al.* (*141*) also applied this method for inventor disambiguation in the USPTO dataset as discussed in the “Semi- and fully supervised classification approaches” section.

### Graphical entity resolution

We now turn to model-based clustering approaches, which allow quantification of uncertainty about the clustering structure. Bhattacharya and Getoor (*163*) built on the latent Dirichlet allocation (LDA) model to this end, where the goal in their application was to resolve individual authors in bibliographic databases. Their approach leveraged coauthorship groups (analogously to topics in LDA) to support the entity resolution process. They probabilistically modeled the unknown set of individual authors, the authors’ group membership, and possible distortions in authors’ names. Posterior inference was carried out using Gibbs sampling.

In a similar spirit, Tancredi and Liseo (*146*) proposed a new model for record linkage, which, instead of linking record to record, linked records to latent individuals. The authors used the hit and miss model of Copas and Hilton (*164*) as a measurement error model to explain possible distortions in the observed data. This deviates from the Fellegi-Sunter approach, as it does not use comparison data, instead working with the actual attribute information.

We refer to such approaches, where one recovers a bipartite graph linking records to reconstructed latent entities, as graphical entity resolution. More specifically, in (*148*) and (*153*), the authors developed a fully hierarchical Bayesian approach to entity resolution, using Dirichlet prior distributions over categorical latent attributes and assuming a data distortion model. They derived an efficient hybrid (Metropolis-within-Gibbs) Markov chain Monte Carlo algorithm for fitting these models. As with other Bayesian approaches, this allows full quantification of uncertainty regarding the number of latent individuals and the clustering structure of records into coreferent groups. In addition, Steorts *et al*. (*153*) showed that, for the proposed work and the work of Sadinle (*30*) and Tancredi and Liseo (*146*), the use of a uniform prior on the set of links or nonlinks, in practice, leads to one having a biased estimation of the sample. This, in turn, led to the development of subjective priors on the linkage structure, which have appeared in (*151*). In addition to Bayesian models for categorical data, Steorts (*149*) extended the above work to both categorical and noisy string data by proposing a string pseudo-likelihood and an empirically motivated prior.

Motivated by the computational limitations of Steorts (*149*) and a case study of the 2010 Census, Marchant *et al*. (*48*) have proposed a scalable extension to this model. Their approach uses probabilistic blocking at the level of the latent entities, which enables distributed inference through a partially collapsed Gibbs sampler while accounting for blocking uncertainty.

### The microclustering property

The work of Steorts (*149*) and Steorts *et al*. (*153*) led to interesting developments both in clustering and in entity resolution. The first is the formalization of the microclustering property, which describes the sublinear growth of clusters in entity resolution (and in other clustering tasks such as community detection). That is, one expects the size of the clusters to grow sublinearly as the total number of records also grows (*151*). On the other hand, traditional probabilistic, generative models for clustering, such as finite mixtures (*165*), Dirichlet processes (*166*–*168*), Pitman-Yor processes (*169*, *170*), and many others, assume a power law growth in the total number of records (data points) (*171*, *172*). Therefore, traditional probabilistic, generative models are misspecified for microclustering tasks, such as entity resolution, given that they have a sublinear growth rate. Therefore, applying a Bayesian nonparametric (BNP) model that favors large clusters makes little sense in the context where each cluster should correspond to a single true entity. The second development is the proposal of general BNP models that can satisfy the microclustering property. The authors also propose a more scalable algorithm, the chaperones algorithm, which allows computational speed-ups for entity resolution that are similar in spirit to the Split and Merge approach as also used by Steorts *et al*. (*153*).

The aforementioned work has led to considerations regarding the feasibility of entity resolution in the context of microclustering. There are only two papers addressing such implications in the literature to our knowledge. In recent work, Steorts *et al*. (*173*) provided the first quantitative bounds that, to our knowledge, can be used for entity resolution. Simulation studies offer guidance for when the bounds are tight and loose in practice. Johndrow *et al*. (*174*) showed that, unless the number of attributes (or features) grows with the number of records, entity resolution is not possible in certain situations.

## DISCUSSION

Here, we have introduced the entity resolution problem as it relates to important social science issues such as the decennial census, human rights violations, voter registration, and inventor and author disambiguation. Applications are more widespread, dealing with medical, housing, and financial databases, among others. We have introduced the main terminology used in the literature, and we have provided the major challenges that researchers face within an entity resolution framework.

We have reviewed deterministic methods (“Deterministic record linkage” section) and seminal probabilistic record linkage methods, such as those proposed by Dunn, Newcombe, and Fellegi and Sunter (“Probabilistic record linkage” section), which led to many modern-day extensions (“Modern probabilistic record linkage” section). These extensions can be viewed as extensions of Fellegi and Sunter (frequentist and Bayesian) or as semi- or fully supervised classification approaches. The “Entity resolution as a clustering problem” section reviewed entity resolution methods that can be viewed as clustering tasks. These include methods where clustering is a post-processing step, graphical entity resolution, and microclustering models.

In the remainder of our discussion, we highlight a few remaining topics that are the subject of active research and that have important practical implications. First, we discuss the need to rigorously evaluate the performance of entity resolution methods in applications. Second, we discuss potential directions regarding scaling Bayesian entity resolution methods. Last, we discuss privacy issues surrounding the use of entity resolution.

### Data fusion, merging, and canonicalization

Here, our focus has been on the structured entity resolution task. Of course, what happens after this task is equally as important, which is in the fourth stage—data fusion, merging, or canonicalization. Specifically, the entity resolution task provides one with potential records that may match to one another; however, it does not tell us which record is the true underlying entity. Canonicalization, merging, or data fusion is the task of merging groups of records that have been classified as matches into one record that represents the true entity (*5*, *175*). The earliest proposals of canonicalization were deterministic, rule-based methods, which were application specific and fast to implement (*177*). The existing literature assumes that training is available to select the canonical record, and authors have proposed optimization and semisupervised methods to finding the most representative record (*178*–*180*). For a full review of data fusion techniques, we refer to the study of Bleiholder and Naumann (*175*). This is an important area of future research as, to our knowledge, it is often unclear how researchers choose the canonical record or canonical dataset and how researchers proceed with downstream tasks, such as logistic regression or predictive analysis.

### Evaluating entity resolution performance

Despite methodological advances, evaluating the performance of entity resolution remains a challenge for a number of reasons. Murray (*181*) expressed concerns regarding overreliance on simple (toy) datasets that may not be representative of real applications, as this could potentially lead methodological research astray. As a starting point, we review in section S2 some public datasets that can be used for comparisons/evaluations. However, given the wide range of fields of application of entity resolution, these datasets are comparatively few in number. We stress that, when using “benchmark datasets,” it is crucial that researchers note the number of records under consideration, the level of noise in the data, its overall quality, and the reliability of the unique identifiers used for performance evaluation. In addition, one should not solely rely on toy datasets, but one should perform carefully thought out simulation studies to understand robustness to model misspecification to provide practitioners with a guide for using their method. In addition, extreme care should be taken regarding sensitivity of tuning parameters in proposed methods and sensitivity of the evaluated performance on choices of such parameters. Last, case studies should be considered, if possible, as this gives one an idea of how proposed methods work for “data in the wild.”

In addition, many researchers advocate the use of expert-labeled data to help train entity resolution model and to evaluate their performance in applications. However, care should be taken as labeling errors and sampling procedures may introduce bias into estimates. Effectively eliciting expert-labeled data while accounting for such sources of bias is an active area of research and one that we consider to be its own field given the complexities involved.

### Scaling entity resolution

Bayesian entity resolution algorithms have been successful in scaling to large datasets, as illustrated by McVeigh *et al*. (*13*) and Marchant *et al.* (*48*). McVeigh *et al.* (*13*) have scaled a Bayesian Fellegi-Sunter approach to roughly 57 millions of records using so-called post hoc blocks. The approach of Marchant *et al.* (*48*) is quite different as blocking and entity resolution are jointly modeled in a Bayesian framework, allowing uncertainty quantification about both parts of the pipeline. The authors scaled to roughly 1 million records using distributed computing. Further research is needed in this area to scale to larger datasets, such as census-size data or industrial-size datasets, while accounting for uncertainties encountered at all stages of the entity resolution pipeline.

### Privacy issues

Entity resolution is fundamentally antithetic to data privacy—it is about gaining information about social entities through the integration of diverse databases. This raises ethical and legal questions for users of entity resolution as well as important privacy considerations (*182*). In particular, as more data are being collected, stored, analyzed, and shared across multiple domains, disclosure risks associated with (even anonymized) data releases become serious. For example, Sweeney (*183*) showed how a simple record linkage algorithm could be used to de-anonymize Netflix movie rankings data through the use of public IMDb profiles. Fienberg and Slavković (*184*) used public voter registration data to de-anonymize a health insurance dataset to showcase the need for stronger privacy measures. These are examples of linkage attacks, where an adversary uses background knowledge (such as voter registration files) to de-anonymize data or to gain information about individuals.

Data releases should therefore be managed through statistical disclosure control (SDC) systems, which aim to balance the utility of released data with privacy protections. To address these competing goals, many SDC techniques have been proposed and implemented such as top-coding, data swapping, data perturbation, and synthetic data generation, each potentially having its own measures of utility and risk properties; more details are those methods that can be found in (*185*, *186*). Furthermore, differential privacy (*187*) has emerged as a key rigorous definition of privacy. It provides a framework that can inform the design of privacy mechanisms with specified disclosure risks, in the presence of arbitrary external information.

As we have discussed, analyses often require or can benefit from the linkage of multiple databases. However, when databases are held by different organizations and contain private information that cannot be shared across them, record linkage should be done to ensure that (i) private information such as quasi-identifiers (name, date of birth, etc.) are not disclosed across organizations during the linkage process and (ii) only relevant summaries of the resulting linkage (usually a set of predetermined attributes of the linked records) are reported to manage disclosure risks. The achievement of these two goals is the subject of privacy-preserving record linkage (PPRL) (*188*–*190*). This is closely related to the problem of private multiparty data publishing under a vertical partitioning scheme (*191*–*194*). While progress has been made on point (i), the privacy implications of post-linkage data releases are difficult to analyze even under mild adversary models (*189*, *192*). Great care should be taken when using PPRL to ensure that all disclosure risks are properly assessed.

### Unstructured and multimodal entity resolution

To our knowledge, one major area that has been explored in the computer science literature, but has been largely unexplored by statisticians, is the task of removing duplications from databases containing unstructured information, such as free-form text and images (*138*, *195*). One application of this is patent databases, which contain unstructured paragraphs (*59*). Another growing application is forensic science, which can involve the matching of fingerprints, ballistics, and other types of evidence from a crime scene (*38*, *39*). In these applications, structured and unstructured data must be combined for accurate linkage and inference. Last, it seems that there is a great need for algorithms that maintain individual-level privacy. However, it is not clear what methods would be most appropriate and if they would work well in practice. For example, are differential privacy measures too stringent? Case studies are needed to understand how such methods could be used in practice.

## Supplementary Materials

### This PDF file includes:

Supplementary Text References
