# Supplementary material for: (Almost) all of entity resolution
Source: Sci Adv. 2022 Mar 25;8(12):eabi8021. doi: 10.1126/sciadv.abi8021 (PMC11636688; doi:10.1126/sciadv.abi8021)
Supplement: Supplementary file 1 — Supplementary Text References [file sciadv.abi8021_sm.pdf]

Science Advances
AAAS

Supplementary Materials for

(Almost) all of entity resolution

Olivier Binette and Rebecca C. Steorts*

*Corresponding author. Email: beka@stat.duke.edu

Published 25 March 2022, *Sci. Adv.* **8**, eabi8021 (2022)
DOI: 10.1126/sciadv.abi8021

This PDF file includes:

Supplementary Text
References

A Open Source Software and Datasets

In this supplement, we review open source entity resolution software (Appendix A.1 and entity resolution datasets (Appendix A.2).

A.1 Open Source Software

This section reviews open source entity resolution software. We focus on libraries available in R or Python software packages, however, we cover a few recent packages that are available in Julia, Java, and Apache Spark. Other software is reviewed in [196, 5, 6].

Available in Python The python library `dedupe` [197], available on PyPI and on GitHub, implements the Fellegi-Sunter framework together with active learning to select threshold weights. Based on this probabilistic record linkage step, it allows clustering records in coreferent groups using hierarchical agglomerative clustering with a centroid linkage. The library `recordlinkage` [198], available on PyPI and on GitHub, implements the Fellegi-Sunter framework, k-means clustering and a number of fully supervised classifiers (logistic regression, support vector machines, etc). The Freely Extensible Biomedical Record Linkage `FEBRL` library [199], available on SourceForge, provides a graphical user interface and implements the Fellegi-Sunter framework as well as supervised classifiers and clustering algorithms. Also, the library `py-entitymatching` [200] (part of the Magellan project [201]), available on PyPI and GitHub, provides tools to facilitate the development of entity resolution models. It implements rule-based systems as well as a number of supervised machine learning classifiers. Finally, the package `fasthash` available on Github implements the work of [54].

Available in R The `RecordLinkage` package on CRAN [202] implements the Fellegi-Sunter framework and a number of supervised algorithms (logistic regression, support vector

machines, random forests, and others). It also contains the two datasets `RLdata500` and `RLdata10000` which have been widely used in the literature as benchmark datasets. [14] extended the work of [95] and provided efficient open source software on CRAN and GitHub known as `fastLink`. The `BRL` package on GitHub implements the bipartite record linkage approach of [31]. The `blink` package on CRAN and GitHub implements the work of [149]. The `fedmatch` package on CRAN implements exact, fuzzy, and probabilistic matching based on the Fellegi-Sunter framework [204].

Available in Julia [13] provide a Julia package to perform blocking and Bayesian Fellegi-Sunter called `BayesianRecordLinkage.jl` on GitHub.

Available in Apache Spark [48] provide a joint blocking and entity resolution package on GitHub, which is provided in Apache Spark with a Java and Scala back-end. The `splink` library [205] implements the Fellegi-Sunter framework in Spark, using the same model as `fastLink`. The `SparkER` library [206] provides an entity resolution framework for Spark.

Available in Java The `Serf` library implements the R-swoosh algorithm [86] for matching and merging. The `JedAI` library [207] provides end-to-end entity resolution with an user-friendly gui.

A.2 Entity Resolution Datasets

In this section, we review entity resolution datasets that are publicly available.

A.2.1 Synthetic datasets

First we review synthetic datasets that are publicly available. For all of these datasets, a unique identifier is available to evaluate entity resolution performance.

RLdata This package contains two synthetic data sets — `RLdata500` and `RLdata10000` from the `RecordLinkage` package in `R`. Attribute information available is first name, last name, and full date of birth.

GeCo Tool One is able to create a synthetic dataset using the `GeCo Tool` [208], where features can consist of first name, last name, and birth date. Distortions can be included as to emulate the effect of optical character recognition, keyboard errors, phonetic errors, and common misspellings.

FEBRL The `FEBRL` datasets [199] consist of comparison patterns from an epidemiological cancer study in Germany (`https://recordlinkage.readthedocs.io/en/latest/ref-datasets.html`).

ABSEmployee The `ABSEmployee` synthetic dataset was constructed to mimic real data from the Australian Bureau of Statistics (ABS), which cannot be released due to privacy reasons. [48] simulated three data sources from the ABS that results in 666,000 total records, with 400,000 unique entities. The three data sources are a supplementary survey of permanent employees (source A), a supplementary survey of all employees (source B), and a census of all employees (source C). The size of source A is 120,000; the size of source B is 180,000; the size of source C is 360,000. Duplication occurs across and within the three data sources.

Feature information available is statistical area level of the employee, mesh block, birth day, birth year, gender (binary), industry, whether employment is on a casual basis (binary), whether employment is full-time, hours worked per week, payrate, average weekly earnings. In all sources, there are missing variables, which are explained further at `https://github.com/cleanzr/dblink-experiments/tree/master/data`.

A.2.2 Real Datasets (Publicly Available)

In this section, we review datasets from the literature which arise from real applications and which are publicly available. For all of these datasets, except for the 1901 and 1911 Irish Census, unique identifiers are available to evaluate entity resolution performance. However, the reliability of these unique identifiers vary. In some cases, these unique identifiers were obtained as the result of extensive record linkage efforts involving expert clerical review of the data. In other cases, the unique identifiers were obtained using external information which is not provided in these datasets.

Cora The `cora` dataset consists of citations and is hosted on the `RIDDLE` repository [209]. Features include title, author, and year of publication. This dataset needs some pre-processing steps before a record linkage method can be applied, such as removing punctuation.

SHIW The Italian Survey on Household and Wealth (FWIW) is a survey that was conducted in 2008 and 2010. Attributes available are branch of activity, employment status, gender, geographical area of birth, highest educational level obtained, town size, year of birth, whether or not Italian national, and working status. The data set can be obtained at `https://github.com/ngmarchant/shiw`.

NLTCS The National Long Term Care Survey (NLTCS) is a publicly available longitudinal survey conducted at Duke University, consisting of six waves. The goal of the survey is to study the health and well being of those older than sixty-five years old across the six waves of the survey. Unfortunately, only three waves are appropriate for record linkage due to issues with the survey design. Thus, only a subset can be utilized, which are waves 1982, 1989 and 1994. The features available for linking are all categorical and are: gender (SEX), full date of birth

(DOB), location of the patient (STATE) and office location of the physician (REGOFF). The provided unique identifier is based upon the social security number. The data is available at `https://www.icpsr.umich.edu/web/NACDA/studies/9681`.

CD The `CD` dataset includes information about 9,763 CDs randomly extracted from freeDB. This dataset can be found at `https://hpi.de/naumann/projects/repeatability/datasets/cd-datasets.html`. There are a total of 299 duplicate records. Attribute information consists of 106 total features such as artist name, title, genre, among others.

Restaurant The `Restaurant` dataset contains duplications of restaurants from Fodor's and Zagat's. Attribute information contains name, address, city, and cuisine.

NCSBE The North Carolina State Board of Elections (NCSBE) releases an online publication of North Carolina voter registration snapshot data. Records are updated temporally, resulting in voters being duplicated within this dataset. While the NCSBE provides each voter with an identifier in each of the snapshots, they do not provide any public information regarding how duplicate records are removed. In addition, the reliability of the NCSBE "unique" voter identifiers has been recently been questioned [114]. Feature information consists of first and last name, age, gender, race, place of birth, age, political affiliation, telephone number, and full address.

USPTO In 2015, PatentsView (see `https://www.patentsview.org/`) organized a competition aiming to develop an inventor disambiguation algorithm for the USPTO patents records. Five datasets of inventor-disambiguated patent records were provided as training data to help develop proposed algorithms and can be downloaded from `https://patentsview.org/events/workshop-2015`.

SDS The `Social Diagnosis Survey (SDS)` is a longitudinal survey regarding households in Poland. The data set is publicly available at `http://www.diagnoza.com/index-en.html`. Feature information available is complete date of birth, gender, residence (province), and level of education.

SIPP The `Survey of Income and Program Participation (SIPP)` is a longitudinal survey of local, state, and federal programs in the United States that collects information about individuals every few years. Specifically, individuals are sampled within panels. The data set is publicly available from the Census Bureau website at `https://www.census.gov/programs-surveys/sipp/data/datasets.html`. Feature information available is year of birth, month of birth, gender, and the state where an individual resides.

1901 and 1911 Irish Census These are two publicly available censuses from Ireland in 1901 and 1911. The census of 1901 occurred on March 31, where the those residing (full name) in a household were recorded in addition to visitors. Addition information was college such as age, gender, relationship to head of household, religion, occupation, marital status, county of birth (unless born abroad, in which case only the country was recorded), ability to read or write, ability to speak Irish, English, both, or none. The census of 1911 was slightly different. The head of household completed and signed the form. In addition, disability status information was collected. Full information on both censuses can be found at `http://www.census.nationalarchives.ie/` and `https://www.irish-genealogy-toolkit.com/census-forms.html`.

A.2.3 Real Sata Sets (Private)

In this section, we review datasets that are not available in the public domain, but have an important place in the literature.

El Salvador Between 1980 and 1991, the Republic of El Salvador witnessed a civil war between the central government, the left-wing guerrilla Farabundo Marti National Liberation Front (FMLN), and right-wing paramilitary death squads. There are three databases available for this conflict, where duplications occur within and across each of the databases. The first two databases were collected *during the conflict*, whereas the third database was collected *after the conflict.* The first two databases contain reports on documented identifiable victims. The first source, **El Rescate** (ER-TL), a nongovernmental organization (based out of Los Angelos, CA), collected electronic data from published reports during the civil war [49]. The second source, **Comission de Derechos Humanos de El Salvador** (CDHES), collected testimonials on violations from 1979 — 1991 [50]. The third source contains reports on documented identifiable victims after the civil war. After the peace agreement in 1992, the United Nations created a **Commission on the Truth** (UNTC), which invited citizens to report war-related human rights violations. As such, victims can be duplicated in these data sets. Further information regarding these datasets is summarized in [31].

Syria One case study that has been of interest is the ongoing Syrian conflict. To our knowledge, the Human Rights Data Analysis Group (HRDAG) provided the first published work in this domain. There are four sources that collected data during the same time period — Syrian Center for Statistics and Research (CSR-SY), Syrian Network for Human Rights (SNHR), Syria Shuhada website (SS), and the the Violation Documentation Centre (VDC). Each source provides documented identifiable deaths in the conflict. Attributes available are full full Arabic name, gender, death location, and date of death. HRDAG has labelled the data set, as outlined in their paper [51].

Decennial Census and Administrative Records One important and timely topic is one that faces the United States Census Bureau each decade when they attempt to count all the individuals

in the population. This enumeration is used to allocate resources for roads, schools, projects, and apportion representation of legislators. Unfortunately, it has been shown difficult to accurate enumerate such a population using an optional census, and response rates are often quite low. Furthermore, some individual may be counted multiple times. For example, an individual that owns three houses might accidentally fill out three census forms. As another example, individuals in group quarters (such as universities, prisons, etc) are often double counted by their "group" and a family member/parent/guardian [47]. De-duplication is thus needed to obtain an accurate enumeration, with new methodology from the machine learning and statistical literature being recently proposed to this end [48]. This methodology is scalable, while providing exact error propagation throughout the blocking and the entity resolution task [48].

California Great Registers Starting in 1900, each country in California (CA) printed and bound voter lists in each election year, which contained the following feature information of each voter: name, address, party registration, and occupation [210, 13]. This became known as the `California Great Registers` dataset, and was used as the county's form of book keeping on election day. These original voter lists have now been digitized using ancestry.com and optical character recognition, however, this can cause errors in the data. The entire dataset can be viewed as a panel dataset, where it may be possible to track partisan change during certain time periods. This dataset spans 1908 — 1968. It is possible to potentially match voters from this time period with individuals from three decennial censuses from 1920, 1930, and 1940, which are publicly available. To our knowledge, the California registers database is not publicly available. Together, the three decennial censuses and the `California Great Registers` dataset combine to form a dataset of 57 million records of Californians.

A.3 Benchmark and Research Data sets for Inventor and Author Disambiguation

In this section, we review some benchmark data sets for author disambiguation that have been recently utilized in the literature.

First we review benchmark data sets that have been used in the literature. **AMINER** contains many author disambiguation data sets used in [211]. As it pertains to this review, the most relevant data set contains author names and ground truth. This data set is publicly available. **Authority 2009** is a Pubmed author data set [212]. **DBLP** is a computer science bibliography data sets, where the full data is publicly available. Groups within the computer science community have created subsets of the DBLP, which are available at PSU-DBLP and Naumann-DBLP. **INSPIRE** is an author disambiguation data set from a digital library for scientific literature in high-energy physics [213]. The data set is publicly available. **Rexa** is a data set on scientific author records derived from bibliographic data, which has been blocked according to unique first initial and last name, which is publicly available. **S2AND** is a union of eight existing author name disambiguation (AND) datasets, described in [36]. Open source software is available.

Now, we review some research data sets, which typically do not have unique identifiers or went through an intensive and well-documented manual labelling process.

KDD 2013 is a challenge data set created by the Microsoft Corporation for author disambiguation, where there are no unique identifiers to our knowledge that are error free. [141] discusses a private data set of 98,762 labeled USPTO records corresponding to inventors of optoelectronics patents. The authors have released various sample pairwise comparisons datasets that are publicly available. The USPTO provides many publicly available research data sets, which have been mostly unexplored from a purely research point of view to our knowledge. **IJCAI 2021** provides papers with authors that have same names, which is publicly available.

REFERENCES AND NOTES

1. A. Doan, A. Halevy, Z. Ives, *Principles of Data Integration* (Morgan Kaufmann Publishers, 2012).

2. A. K. Elmagarmid, P. G. Ipeirotis, V. S. Verykios, Duplicate record detection: A survey. *IEEE Trans. Knowledge Data Eng.* **19**, 1–16 (2007).

3. F. Naumann, M. Herschel, *An Introduction to Duplicate Detection* (Morgan & Claypool Publishers, 2010).

4. L. Getoor, A. Machanavajjhala, Entity resolution: Theory, practice & open challenges. *Proc. VLDB Endowment* **5**, 2018–2019 (2012).

5. P. Christen, *Data Matching: Concepts and Techniques for Record Linkage, Entity Resolution, and Duplicate Detection* (Data-Centric Systems and Applications, Springer-Verlag, 2012).

6. V. Christophides, V. Efthymiou, T. Palpanas, G. Papadakis, K. Stefanidis, An overview of end-to-end entity resolution for big data. *ACM Computing Surveys* **53**, 1–42 (2021).

7. I. F. Ilyas, X. Chu, *Data Cleaning* (Association for Computing Machinery, 2019).

8. G. Papadakis, E. Ioannou, E. Thanos, T. Palpanas, *The Four Generations of Entity Resolution* (Morgan & Claypool Publishers, 2021).

9. T. Herzog, F. Scheuren, W. Winkler, *Data Quality and Record Linkage Techniques* (Springer, 2007).

10. W. E. Winkler, Matching and record linkage. *Wiley Interdiscip. Rev. Comput. Stat.* **6**, 313–325 (2014).

11. A. Jurek-Loughrey, P. Deepak, in *Semi-Supervised and Unsupervised Approaches to Record Pairs Classification in Multi-Source Data Linkage* (Springer, 2019), pp. 55–78.

12. J. Asher, D. Resnick, J. Brite, R. Brackbill, J. Cone, An introduction to probabilistic record linkage with a focus on linkage processing for wtc registries. *Int. J. Environ. Res. Public Health* **17**, 6937 (2020).

13. B. S. McVeigh, B. T. Spahn, J. S. Murray, Scaling Bayesian probabilistic record linkage with post-hoc blocking: An application to the california great registers. arXiv:1905.05337 [stat.ME] (14 May 2019).

14. T. Enamorado, B. Fifield, K. Imai, Using a probabilistic model to assist merging of large-scale administrative records. *Am. Polit. Sci. Rev.* **113**, 353–371 (2019).

15. E. Rogot, P. Sorlie, N. J. Johnson, Probabilistic methods in matching census samples to the National Death Index. *J. Chronic Dis.* **39**, 719–734 (1986).

16. N. Méray, J. B. Reitsma, A. C. Ravelli, G. J. Bonsel, Probabilistic record linkage is a valid and transparent tool to combine databases without a patient identification number. *J. Clin. Epidemiol.* **60**, 883–891 (2007).

17. M. A. Jaro, Probabilistic linkage of large public health data files. *Stat. Med.* **14**, 491–498 (1995).

18. R. Gutman, C. C. Afendulis, A. M. Zaslavsky, A Bayesian procedure for file linking to analyze end-of-life medical costs. *J. Am. Stat. Assoc.* **108**, 34–47 (2013).

19. M. Shan, K. Thomas, R. Gutman, A Bayesian multi-layered record linkage procedure to analyze functional status of medicare patients with traumatic brain injury. arXiv:2005.08549 [stat.ME] (18 May 2020).

20. E. Farley, R. Gutman, A Bayesian approach to linking data without unique identifiers. arXiv:2012.00601 [stat.CO] (1 December 2020).

21. M. A. Jaro, Advances in record-linkage methodology as applied to matching the 1985 census of Tampa, Florida. *J. Am. Stat. Assoc.* **84**, 414–420 (1989).

22. W. E. Winkler, Y. Thibaudeau, *An Application of the Fellegi-Sunter Model of Record Linkage to the 1990 US Decennial Census* (U.S. Census Bureau, 1990), pp. 1–22.

23. M. Fortini, B. Liseo, A. Nuccitelli, M. Scanu, On Bayesian record linkage. *Res. Official Stat.* **4**, 185–198 (2001).

24. A. Chevrette, “G-link: A probabilistic record linkage system” (Technical Report, Statistics Canada, 2011).

25. A. Dasylva, R.-C. Titus, C. Thibault, Overcoverage in the 2011 Canadian census, in *Proceedings of Statistics Canada Symposium* (Statistics Canada, 2014).

26. A. Dasylva, Pairwise estimating equations for the primary analysis of linked data, in *Proceedings of Statistics Canada Symposium* (Statistics Canada, 2018).

27. K. Lum, M. E. Price, D. Banks, Applications of multiple systems estimation in human rights research. *Am. Statist.* **67**, 191–200 (2013).

28. M. Price, A. Gohdes, P. Ball, Documents of war: Understanding the Syrian conflict. *Significance* **12**, 14–19 (2015).

29. P. Sadosky, A. Shrivastava, M. Price, R. C. Steorts, Blocking methods applied to casualty records from the Syrian conflict. arXiv:1510.07714 [stat.AP] (26 October 2015).

30. M. Sadinle, Detecting duplicates in a homicide registry using a Bayesian partitioning approach. *Annal. Appl. Stat.* **8**, 2404–2434 (2014).

31. M. Sadinle, Bayesian estimation of bipartite matchings for record linkage. *J. Am. Stat. Assoc.* **112**, 600–612 (2017).

32. M. Sadinle, Bayesian propagation of record linkage uncertainty into population size estimation of human rights violations. *Annal. Appl. Stat.* **12**, 1013–1038 (2018).

33. R. Lai, A. D’amour, A. Yu, Y. Sun, L. Fleming, *Disambiguation and Co-Authorship Networks of the US Patent Inventor Database (1975–2010)* (Harvard Institute for Quantitative Social Science, 2011), vol. 2138.

34. G. Louppe, H. T. Al-Natsheh, M. Susik, E. J. Maguire, Ethnicity sensitive author disambiguation using semi-supervised learning, in *Proceedings of the International Conference on Knowledge Engineering and the Semantic Web* (Springer, 2016), pp. 272–287.

35. Y. Zhang, F. Zhang, P. Yao, J. Tang, Name disambiguation in aminer: Clustering, maintenance, and human in the loop, in *Proceedings of the 24th ACM SIGKDD International Conference on Knowledge Discovery & Data Mining* (Association for Computing Machinery, 2018), pp. 1002–1011.

36. S. Subramanian, D. King, D. Downey, S. Feldman, S2AND: A benchmark and evaluation system for author name disambiguation. arXiv:2103.07534 [cs.DL] (12 March 2021).

37. X. Liu, D. Yin, X. Zhang, K. Su, K. Wu, H. Yang, J. Tan, OAG-BERT: Pre-train heterogeneous entity-augmented academic language models. arXiv:2103.02410 [cs.CL] (3 March 2021).

38. X. H. Tai, Record linkage and matching problems in forensics, in *Proceedings of the IEEE International Conference on Data Mining Workshops* (IEEE, 2018), pp. 510–517.

39. X. H. Tai, W. F. Eddy, Automatically matching topographical measurements of cartridge cases using a record linkage framework. arXiv:2003.00060 [stat.AP] (28 February 2020).

40. W. Fan, X. Jia, J. Li, S. Ma, Reasoning about record matching rules. *Proc. VLDB Endowment* **2**, 407–418 (2009).

41. R. Singh, V. Meduri, A. Elmagarmid, S. Madden, P. Papotti, J.-A. Quiane-Riuz, A. Solar-Lezama, N. Tang, Generating concise entity matching rules, in *Proceedings of the 2017 ACM International Conference on Management of Data* (Association for Computing Machinery, 2017), pp. 1635–1638.

42. S. Mudgal, H. Li, T. Rekatsinas, A. Doan, Y. Park, G. Krishnan, R. Deep, E. Arcaute, V. Raghavendra, Deep learning for entity matching: A design space exploration, in *Proceedings of the 2018 International Conference on Management of Data* (Association for Computing Machinery, 2018), pp. 19–34.

43. J. Wang, T. Kraska, M. J. Franklin, J. Feng, Crowder: Crowdsourcing entity resolution. *Proc. VLDB Endowment* **5**, 1483–1494 (2012).

44. C. Gokhale, S. Das, A. Doan, J. F. Naughton, N. Rampalli, J. Shavlik, X. Zhu, *Corleone: Hands-off Crowdsourcing for Entity Matching* (Association for Computing Machinery, 2014), pp. 601–612.

45. G. Papadakis, J. Svirsky, A. Gal, T. Palpanas, Comparative analysis of approximate blocking techniques for entity resolution. *Proc. VLDB Endowment* **9**, 684–695 (2016).

46. H. L. Dunn, Record linkage. *Am. J. Public Health Nations Health* **36**, 1412–1416 (1946).

47. H. Hogan, P. J. Cantwell, J. Devine, V. T. Mule, V. Velkoff, Quality and the 2010 census. *Population Res. Policy Rev.* **32**, 637–662 (2013).

48. N. G. Marchant, R. C. Steorts, A. Kaplan, B. I. P. Rubinstein, D. N. Elazar, d-blink: Distributed end-to-end Bayesian entity resolution. arXiv:1909.06039 [stat.CO] (13 September 2019).

49. T. Howland, How El Rescate, a small nongovernmental organization, contributed to the transformation of the human rights situation in El Salvador. *Hum. Rights Q.* **30**, 703–757 (2008).

50. P. Ball, The Salvadoran human rights commission: Data processing, data representation, and generating analytical reports, in *Making the Case: Investigating Large Scale Human Rights Violations Using Information Systems and Data Analysis*, P. Ball, H. F. Spirer, L. Spirer, Eds. (American Association for the Advancement of Science, 2000), pp. 15–24.

51. M. Price, J. Klingner, A. Qtiesh, P. Ball, Full updated statistical analysis of documentation of killing in the Syrian Arab Repulic, in *Report by the Human Rights Data Analysis Group to the United Nations Office of the High Commissioner for Human Rights (OHCHR)* (Office of the UN High Commissioner for Human Rights, 2013).

52. M. Price, P. Ball, Big data, selection bias, and the statistical patterns of mortality in conflict. *SAIS Rev. Int. Aff.* **34**, 9–20 (2014).

53. A. H. Green, P. Ball, Civilian killings and disappearances during civil war in El Salvador (1980–1992). *Demogr. Res.* **41**, 781–814 (2019).

54. B. Chen, A. Shrivastava, R. C. Steorts, Unique entity estimation with application to the Syrian conflict. *Annal. Appl. Stat.* **12**, 1039–1067 (2018).

55. J. Ax, Georgia lawsuit is latest blow in U.S. fight over voting rights (2018) [posted 12 October 2018; retrieved 17 July 2020].

56. B. Nadler, Voting rights become a flashpoint in georgia governor's race (2018) [posted 9 October 2018; retrieved 17 July 2020].

57. T. Enamorado, Georgia's 'exact match' law could potentially harm many eligible voters (2018) [posted 20 October 2018; retrieved 17 July 2020].

58. *Georgia Coalition For the Peoples' Agenda Inc. et al.* v. *Kemp*, Complaint for injunctive and declaratory relief (2018).

59. G. C. Li, R. Lai, A. D'Amour, D. M. Doolin, Y. Sun, V. I. Torvik, A. Z. Yu, L. Fleming, Disambiguation and co-authorship networks of the U.S. patent inventor database (1975–2010). *Res. Policy* **43**, 941–955 (2014).

60. M.-C. Müller, F. Reitz, N. Roy, Data sets for author name disambiguation: An empirical analysis and a new resource. *Scientometrics* **111**, 1467–1500 (2017).

61. X. L. Dong, D. Srivastava, *Big Data Integration* (Morgan and Claypool Publishers, 2015).

62. V. I. Levenshtein, Binary codes capable of correcting deletions, insertions, and reversals. *Soviet Phys. Doklady* **10**, 707–710 (1966).

63. L. Yujian, L. Bo, A normalized levenshtein distance metric. *IEEE Trans. Pattern Anal. Mach. Intell.* **29**, 1091–1095 (2007).

64. W. E. Winkler, String comparator metrics and enhanced decision rules in the Fellegi-Sunter model of record linkage, in *Proceedings of the Section on Survey Research, American Statistical Association* (American Statistical Association, 1990), pp. 354–359.

65. G. Navarro, A guided tour to approximate string matching. *ACM Comput. Surveys* **33**, 31–88 (2001).

66. W. W. Cohen, P. Ravikumar, S. E. Fienberg, A comparison of string distance metrics for name-matching tasks, in *Proceedings of the 2003 International Conference on Information Integration on the Web* (AAAI Press, 2003), pp. 73–78.

67. J. Wang, G. Li, J. X. Yu, J. Feng, Entity matching: How similar is similar. *Proc. VLDB Endowment* **4**, 622–633 (2011).

68. C. R. Rivero, D. Ruiz, Selecting suitable configurations for automated link discovery, in *Proceedings of the ACM Symposium on Applied Computing* (Association for Computing Machinery, 2020), pp. 907–914.

69. E. Ristad, P. Yianilos, Learning string-edit distance. *IEEE Trans. Pattern Anal. Mach. Intell.* **20**, 522–532 (1998).

70. H. Galhardas, D. Florescu, D. Shasha, E. Simon, C. Saita, "Declarative data cleaning: Language, model, and algorithms," thesis, INRIA (2001).

71. M. Bilenko, R. J. Mooney, Adaptive duplicate detection using learnable string similarity measures, in *Proceedings of the Ninth ACM SIGKDD International Conference on Knowledge Discovery and Data Mining* (Association for Computing Machinery, 2003), pp. 39–48.

72. A. McCallum, K. Bellare, F. Pereira, A conditional random field for discriminatively-trained finite-state string edit distance. arXiv:1207.1406 [cs.LG] (4 July 2012).

73. M. Nentwig, M. Hartung, A. C. Ngonga Ngomo, E. Rahm, A survey of current link discovery frameworks. *Semantic Web* **8**, 419–436 (2016).

74. N. Andrews, J. Eisner, M. Dredze, Name phylogeny: A generative model of string variation, in *Proceedings of the 2012 Joint Conference on Empirical Methods in Natural Language Processing and Computational Natural Language Learning* (Association for Computational Linguistics, 2012), pp. 344–355.

75. W. W. Cohen, Data integration using similarity joins and a word-based information representation language. *ACM Trans. Inform. Syst.* **18**, 288–321 (2000).

76. T. Soru, A. C. N. Ngomo, Rapid execution of weighted edit distances. *Proc. Ontol. Matching Workshop* **1111**, 1–12 (2013).

77. H. Zhang, Q. Zhang, Embedjoin: Efficient edit similarity joins via embeddings, in *Proceedings of the ACM SIGKDD International Conference on Knowledge Discovery and Data Mining* (Association for Computing Machinery, 2017), pp. 585–594.

78. J. Wang, J. Feng, G. Li, Trie-join: Efficient triebased string similarity joins with edit distance constraints. *Proc. VLDB Endowment* **3**, 1219–1230 (2010).

79. M. Yu, J. Wang, G. Li, Y. Zhang, D. Deng, J. Feng , A unified framework for string similarity search with edit-distance constraint. *VLDB J.* **26**, 249–274 (2017).

80. H. Wei, J. X. Yu, C. Lu, String similarity search: A hash-based approach. *IEEE Trans. Knowl. Data Eng.* **30**, 170–184 (2018).

81. G. Papadakis, D. Skoutas, E. Thanos, T. Palpanas, Blocking and filtering techniques for entity resolution: A survey. *ACM Comput. Surveys* **53**, 1–42 (2020).

82. A. E. Monge, C. P. Elkan, An efficient domain-independent algorithm for detecting approximately duplicate database records, in *Proceedings of the SIGMOD 1997 Workshop on Research Issues on Sata Mining and Knowledge Discovery* (DMKD, 1997), pp. 23–29.

83. O. Hassanzadeh, F. Chiang, H. C. Lee, R. J. Miller, Framework for evaluating clustering algorithms in duplicate detection. *Proc. VLDB Endowment* **2**, 1282–1293 (2009).

84. A. Saeedi, E. Peukert, E. Rahm, in *Comparative Evaluation of Distributed Clustering Schemes for Multi-source Entity Resolution* (Springer International Publishing, 2017), pp. 278–293.

85. A. Heidari, G. Michalopoulos, S. Kushagra, I. F. Ilyas, T. Rekatsinas, Record fusion: A learning approach. arXiv:2006.10208 [cs.LG] (18 June 2020).

86. O. Benjelloun, H. Garcia-Molina, D. Menestrina, Q. Su, S.E. Whang, J. Widom , Swoosh: A generic approach to entity resolution. *VLDB J.* **18**, 255–276 (2009).

87. S. B. Dusetzina, S. Tyree, A.M. Meyer, A. Meyer, L. Green, W.R. Carpenter, *Linking Data for Health Services Research: A Framework and Instructional Guide* (Agency for Healthcare Research and Quality, 2014).

88. S. Gomatam, R. Carter, M. Ariet, G. Mitchell, An empirical comparison of record linkage procedures. *Stat. Med.* **21**, 1485–1496 (2002).

89. K. M. Campbell, D. Deck, A. Krupski, Record linkage software in the public domain: A comparison of Link plus, the Link King, and a 'basic' deterministic algorithm. *Health Informatics J.* **14**, 5–15 (2008).

90. M. Tromp, A. C. Ravelli, G. J. Bonsel, A. Hasman, J. B. Reitsma, Results from simulated data sets: Probabilistic record linkage outperforms deterministic record linkage. *J. Clin. Epidemiol.* **64**, 565–572 (2011).

91. T. Avoundjian, J.C. Dombrowski, M.R. Golden, J.P. Hughes, B.L. Guthrie, J. Baseman, M. Sadinle , Comparing methods for record linkage for public health action: Matching algorithm validation study. *JMIR Public Health Surveill.* **6**, e15917 (2020).

92. R. C. Steorts, S. L. Ventura, M. Sadinle, S. E. Fienberg, A comparison of blocking methods for record linkage, in *Privacy in Statistical Databases*, J. Domingo-Ferrer, Ed. (Springer, 2014), pp. 253–268.

93. J. S. Murray, Probabilistic record linkage and deduplication after indexing, blocking, and filtering. *J. Privacy Confidential.* **7**, 3–24 (2016).

94. H. B. Newcombe, J. M. Kennedy, S. J. Axford, A. P. James, Automatic linkage of vital records. *Science* **130**, 954–959 (1959).

95. I. P. Fellegi, A. B. Sunter, A theory for record linkage. *J. Am. Stat. Assoc.* **64**, 1183–1210 (1969).

96. R. Wu, S. Chaba, S. Sawlani, X. Chu, S. Thirumuruganathan, ZeroER: Entity resolution using zero labeled examples, in *Proceedings of the 2020 ACM SIGMOD International Conference on Management of Data* (Association for Computing Machinery, 2020), pp. 1149–1164.

97. H. B. Newcombe, P. O. W. Rhynas, Child spacing following stillbirth and infant death. *Eugen. Q.* **9**, 25–35 (1962).

98. H. B. Newcombe, The study of mutation and selection in human populations. *Eugen. Rev.* **57**, 109–125 (1965).

99. H. B. Newcombe, O. G. Tavendale, Effects of father's age on the risk of child handicap or death. *Obstet. Gynecol. Survey* **20**, 655–656 (1965).

100. H. B. Newcombe, Couplage de donnees pour les etudes demographiques. *Population* **24**, 653 (1969).

101. W. E. Winkler, Using the EM algorithm for weight computation in the Fellegi-Sunter model of record linkage, in *Proceedings of the Section on Survey Research Methods* (American Statistical Association, 1988), pp. 667–671.

102. M. D. Larsen, D. B. Rubin, Iterative automated record linkage using mixture models. *J. Am. Stat. Assoc.* **96**, 32–41 (2001).

103. M. E. Smith, H. B. Newcombe, Methods for computer linkage of hospital admission separation records into cumulative health histories. *Methods Inf. Med.* **14**, 118–125 (1975).

104. Y. Thibaudeau, The discrimination power of dependency structures in record linkage. *Survey Methodol.* **19**, (1993).

105. J. Armstrong, J. Mayda, Estimation of record linkage models using dependent data, in *Proceedings of the Section on Survey Research Methodology* (American Statistical Association, 1992), pp. 853–858.

106. W. E. Winkler, Comparative analysis of record linkage decision rules, in *Proceedings of the Section on Survey Research Methods* (American Statistical Association, 1992), pp. 829–834.

107. W. E. Winkler, Improved decision rules in the Fellegi-Sunter model of record linkage, in *Proceedings of the Section on Survey Research Methods* (American Statistical Association, 1993), pp. 274–279.

108. T. R. Belin, D. B. Rubin, A method for calibrating false-match rates in record linkage. *J. Am. Stat. Assoc.* **90**, 694–707 (1995).

109. T. R. Belin, A proposed improvement in computer matching techniques, in *Statistics of Income and Related Administrative Record Research* (International Revenue Service, 1990), pp. 167–172.

110. K. Nigam, A. K. McCallum, S. Thrun, T. Mitchell, Text classification from labeled and unlabeled documents using EM. *Mach. Learn.* **39**, 103–134 (2000).

111. W. E. Winkler, Machine learning, information retrieval, and record linkage, in *Proceedings of the Section on Survey Research Methods* (American Statistical Association, 2000), pp. 20–29.

112. W. E. Winkler, "Methods for record linkage and Bayesian networks" (Technical Report, Statistical Research Division, U.S. Census Bureau, 2002).

113. P. Lahiri, M. Larsen, Regression analysis with linked data. *J. Am. Stat. Assoc.* **100**, 222–230 (2005).

114. J. P. H. Wortman, "Record linkage methods with applications to causal inference and election voting data," thesis, Duke University (2019).

115. O. Chapelle, S. Bernhard, A. Zien, *Semi-Supervised Learning* (The MIT Press, 2006).

116. T. Enamorado, *Active Learning for Probabilistic Record Linkage* (Princeton University, 2019).

117. S. Sarawagi, A. Bhamidipaty, Interactive deduplication using active learning, in *Proceedings of the ACM SIGKDD International Conference on Knowledge Discovery and Data Mining* (Association for Computing Machinery, 2002), pp. 269–278.

118. K. Bellare, S. Iyengar, A. G. Parameswaran, V. Rastogi, Active sampling for entity matching, in *Proceedings of the 18th ACM SIGKDD International Conference on Knowledge Discovery and Data Mining* (2012), pp. 1131–1139.

119. Q. Wang, D. Vatsalan, P. Christen, Efficient interactive training selection for large-scale entity resolution, in *Advances in Knowledge Discovery and Data Mining* (Springer, 2015), pp. 562–573.

120. P. Christen, D. Vatsalan, Q. Wang, Efficient entity resolution with adaptive and interactive training data selection, in *Proceedings of the IEEE International Conference on Data Mining* (IEEE, 2015), pp. 727–732.

121. D. Firmani, B. Saha, D. Srivastava, Online entity resolution using an oracle. *Proc. VLDB Endowment* **9**, 384–395 (2016).

122. M. Kejriwal, D. P. Miranker, Semi-supervised instance matching using boosted classifiers, in *Proceedings of the European Semantic Web Conference* (Springer, 2015), pp. 388–402.

123. N. Vesdapunt, K. Bellare, N. Dalvi, Crowdsourcing algorithms for entity resolution. *Proc. VLDB Endowment* **7**, 1071–1082 (2014).

124. K. Frisoli, B. LeRoy, R. Nugent, A novel record linkage interface that incorporates group structure to rapidly collect richer labels, in *Proceedings of the IEEE International Conference on Data Science and Advanced Analytics* (IEEE, 2019), pp. 580–589.

125. M. Trajtenberg, G. Shiff, "Identification and mobility of Israeli patenting inventors" (Technical Report, Pinhas Sapir Center for Development, 2008).

126. P. Azoulay, J. S. G. Zivin, B. N. Sampat, The diffusion of scientific knowledge across time and space: Evidence from professional transitions for the superstars of medicine, *The Rate and Direction of Inventive Activity Revisited*, J. Lerner, S. Stern, Eds. (University of Chicago Press, 2012).

127. M. J. Bailey, C. Cole, M. Henderson, C. Massey, How well do automated linking methods perform? Lessons from US historical data. *J. Econ. Lit.* **58**, 997–1044 (2020).

128. V. I. Torvik, M. Weeber, D. R. Swanson, N. R. Smalheiser, A probabilistic similarity metric for medline records: A model for author name disambiguation. *J. Am. Soc. Inform. Sci. Technol.* **56**, 140–158 (2005).

129. P. Christen, A two-step classification approach to unsupervised record linkage, in *Proceedings of the Sixth Australasian Conference on Data Mining and Analytics* (2007), pp. 111–119.

130. P. Christen, Automatic record linkage using seeded nearest neighbour and support vector machine classification, in *Proceedings of the 14th ACM SIGKDD International Conference on Knowledge Discovery and Data Mining* (Association for Computing Machinery, 2008), pp. 151–159.

131. R. D. Gottapu, C. Dagli, B. Ali, Entity resolution using convolutional neural network. *Procedia Comput. Sci.* **95**, 153–158 (2016).

132. M. Ebraheem, S. Thirumuruganathan, S. Joty, M. Ouzzani, N. Tang, DeepER–Deep entity resolution, arXiv:1710.00597 [cs.DB] (2 October 2017).

133. M. Ebraheem, S. Thirumuruganathan, S. Joty, M. Ouzzani, N. Tang, Distributed representations of tuples for entity resolution. *Proc. VLDB Endowment* **11**, 1454–1467 (2018).

134. N. Kooli, R. Allesiardo, E. Pigneul, Deep learning based approach for entity resolution in databases, in *Intelligent Information and Database Systems* (Springer International Publishing, 2018), pp. 3–12.

135. J. Kasai, K. Qian, S. Gurajada, Y. Li, L. Popa, Low-resource deep entity resolution with transfer and active learning, in *Proceedings of the 57th Annual Meeting of the Association for Computational Linguistics* (Association for Computational Linguistic, 2020), pp. 5851–5861.

136. N. Barlaug, J. A. Gulla, Neural networks for entity matching: A survey. *ACM Trans. Knowledge Discov. Data* **15**, 1–37 (2021).

137. B. Li, W. Wang, Y. Sun, L. Zhang, M. A. Ali, Y. Wang, GraphER: Token-centric entity resolution with graph convolutional neural networks, in *Proceedings of the 34th AAAI Conference on Artificial Intelligence* (AAAI, 2020), pp. 8172–8179.

138. Y. Li, J. Li, Y. Suhara, J. Wang, W. Hirota, W. C. Tan, Deep entity matching: Challenges and opportunities. *J. Data Inform. Quality* **13**, 1–17 (2021).

139. Y. Li, J. Li, Y. Suhara, A. Doan, W. C. Tan, Deep entity matching with pre-trained language models. *Proc. VLDB Endowment* **14**, 50–60 (2020).

140. T. Hastie, R. Tibshirani, J. Friedman, *The Elements of Statistical Learning: Data Mining, Inference, and Prediction* (Springer, 2001).

141. S. L. Ventura, R. Nugent, E. R. Fuchs, Seeing the non-stars: (Some) sources of bias in past disambiguation approaches and a new public tool leveraging labeled records. *Res. Policy* **44**, 1672–1701 (2015).

142. P. Azoulay, R. Michigan, B. N. Sampat, The anatomy of medical school patenting. *N. Eng. J. Med.* **357**, 2049–2056 (2007).

143. L. Fleming, C. King III, A. I. Juda, Small worlds and regional innovation. *Organization Sci.* **18**, 938–954 (2007).

144. A. Arasu, M. Götz, R. Kaushik, On active learning of record matching packages, in *Proceedings of the 2010 ACM SIGMOD International Conference on Management of data* (Association for Computing Machinery, 2010), pp. 783–794.

145. W. W. Cohen, J. Richman, Learning to match and cluster large high-dimensional data sets for data integration, in *Proceedings of the Eighth ACM SIGKDD International Conference on Knowledge Discovery and Data Mining* (Association for Computing Machinery, 2002), pp. 475–480.

146. A. Tancredi, B. Liseo, A hierarchical Bayesian approach to record linkage and population size problems. *Annal. Appl. Stat.* **5**, 1553–1585 (2011).

147. S. L. Ventura, R. Nugent, E. R. Fuchs, Hierarchical linkage clustering with distributions of distances for large scale record linkage, in *Privacy in Statistical Databases*, J. Domingo-Ferrer, Ed. (Springer, 2014), pp. 283–298.

148. R. C. Steorts, R. Hall, S. E. Fienberg, SMERED: A Bayesian approach to graphical record linkage and de-duplication. *J. Mach. Learn. Res.* **33**, 922–930 (2014).

149. R. C. Steorts, Entity resolution with empirically motivated priors. *Bayesian Anal.* **10**, 849–875 (2015).

150. E. Rahm, The case for holistic data integration, in *Advances in Databases and Information Systems* (Springer International Publishing, 2016), pp. 11–27.

151. G. Zanella, B. Betancourt, H. Wallach, J. Miller, A. Zaidi, R. C. Steorts, Flexible models for microclustering with application to entity resolution, in *Proceedings of the 30th International Conference on Neural Information Processing Systems* (2016), pp. 1425–1433.

152. N. Monath, A. Kobren, A. Krishnamurthy, M. R. Glass, A. McCallum, Scalable hierarchical clustering with tree grafing, in *Proceedings of the ACM SIGKDD International Conference on Knowledge Discovery and Data Mining* (Association for Computing Machinery, 2019), pp. 1438–1448.

153. R. C. Steorts, R. Hall, S. E. Fienberg, A Bayesian approach to graphical record linkage and deduplication. *J. Am. Stat. Assoc.* **111**, 1660–1672 (2016).

154. J. Han, M. Kamber, J. Pei, *Data Mining: Concepts and Techniques* (Morgan Kaufmann Publishers, 2011).

155. M. A. Hernández, S. J. Stolfo, The merge/purge problem for large databases, in *Proceedings of the 1995 ACM SIGMOD International Conference on Management of Data* (Association for Computing Machinery, 1995), pp. 127–138.

156. M. A. Hernández, S. J. Stolfo, Real-world data is dirty: Data cleansing and the merge/purge problem. *Data Mining Knowledge Discov.* **2**, 9–37 (1998).

157. N. Bansal, A. Blum, S. Chawla, Correlation clustering. *Mach. Learn.* **56**, 89–113 (2004).

158. V. Filkov, S. Skiena, Integrating microarray data by consensus clustering, in *Proceedings of the International Conference on Tools with Artificial Intelligence* (ICTAI, 2003), pp. 418–426.

159. M. Charikar, V. Guruswami, A. Wirth, Clustering with qualitative information. *J.Comput. Syst. Sci.* **71**, 360–383 (2005).

160. N. Ailon, M. Charikar, A. Newman, Aggregating inconsistent information: Ranking and clustering. *J. Assoc. Comput. Mach.* **55**, 1–27 (2008).

161. A. Gionis, H. Mannila, P. Tsaparas, Clustering aggregation. *ACM Trans. Knowledge Discov. Data* **1**, 4-es (2007).

162. S. C. Johnson, Hierarchical clustering schemes. *Psychometrika* **32**, 241–254 (1967).

163. I. Bhattacharya, L. Getoor, A latent dirichlet model for unsupervised entity resolution, in *Proceedings of the Sixth SIAM International Conference on Data Mining* (Society for Industrial and Applied Mathematics, 2006), pp. 47–58.

164. J. B. Copas, F. J. Hilton, Record linkage: Statistical models for matching computer records. *J. R. Stat. Soc. A* **153**, 287–320 (1990).

165. D. M. Blei, A. Y. Ng, M. I. Jordan, Latent Dirichlet allocation. *J. Mach. Learn. Res.* **3**, 993–1022 (2003).

166. C. E. Antoniak, Mixtures of Dirichlet processes with applications to Bayesian nonparametric problems. *Annal. Stat.* **2**, 1152–1174 (1974).

167. S. N. MacEachern, Estimating normal means with a conjugate style Dirichlet process prior. *Commun. Stat. Simul. Comput.* **23**, 727–741 (1994).

168. S. N. MacEachern, Computational methods for mixture of Dirichlet process models, in *Practical Nonparametric and Semiparametric Bayesian Statistics* (Springer, 1998), pp. 23–43.

169. M. Perman, J. Pitman, M. Yor, Size-biased sampling of Poisson point processes and excursions. *Probability Theory Related Fields* **92**, 21–39 (1992).

170. J. Pitman, M. Yor, The two-parameter Poisson-Dirichlet distribution derived from a stable subordinator. *Annal. Probability* **25**, 855–900 (1997).

171. J. F. C. Kingman, The representation of partition structures. *J. Lond. Math. Soc.* **s2-18**, 374–380 (1978).

172. T. Broderick, J. Pitman, M. I. Jordan, Feature allocations, probability functions, and paintboxes. *Bayesian Anal.* **8**, 801–836 (2013).

173. R. C. Steorts, M. Barnes, W. Neiswanger, Performance bounds for graphical record linkage, in *Proceedings of the 20th International Conference on Artificial Intelligence and Statistics* (2017), vol. 54, pp. 298–306.

174. J. E. Johndrow, K. Lum, D. B. Dunson, Theoretical limits of microclustering for record linkage. *Biometrika* **105**, 431–446 (2018).

175. J. Bleiholder, F. Naumann, Data fusion. *ACM Comput. Surv.* **41**, 1–41 (2009).

176. S. Cohen, Y. Sagiv, An incremental algorithm for computing ranked full disjunctions, in *Proceedings of the Twenty-Fourth ACM SIGMOD-SIGACT-SIGART Symposium on Principles of Database Systems* (Association for Computing Machinery, 2005), pp. 98–107.

177. L. L. Yan, M. T. Ozsu, Conflict tolerant queries in aurora, in *Proceedings Fourth IFCIS International Conference on Cooperative Information Systems* (IEEE, 1999), pp. 279–290.

178. P. Bohannon, W. Fan, M. Flaster, R. Rastogi, A cost-based model and effective heuristic for repairing constraints by value modification, in *Proceedings of the 2005 ACM SIGMOD International Conference on Management of Data* (Association for Computing Machinery, 2005), pp. 143–154.

179. A. Culotta, M. Wick, R. Hall, M. Marzilli, A. McCallum, Canonicalization of database records using adaptive similarity measures, in *Proceedings of the 13th ACM SIGKDD International Conference on Knowledge Discovery and Data Mining* (Association for Computing Machinery, 2007), pp. 201–209.

180. J. Murray, A unified framework for de-duplication and population size estimation (invited discussion). *Bayesian Anal.* **15**, 664–669 (2020).

181. J. Lane, V. Stodden, S. Bender, H. Nissenbaum, *Privacy, Big Data, and the Public Good: Frameworks for Engagement* (Cambridge Univ. Press, 2014).

182. A. Narayanan, V. Shmatikov, Robust de-anonymization of large sparse datasets, in *Proceedings of the IEEE Symposium on Security and Privacy* (IEEE, 2008), pp. 111–125.

183. L. Sweeney, K-anonymity: A model for protecting privacy. *Int. J. Uncertain. Fuzziness Knowl-Based Syst.* **10**, 557–570 (2002).

184. S. Fienberg, A. Slavković, in *Data Privacy and Confidentiality* (International Encyclopedia of Statistical Science, Springer-Verlag, 2011), pp. 342–345.

185. A. Hundepool, J. Domingo-Ferrer, L. Franconi, S. Giessing, E. S. Nordholt, K. Spicer, P. P. de Wolf, *Statistical Disclosure Control* (John Wiley & Sons, 2012).

186. C. Dwork, F. McSherry, K. Nissim, A. Smith, Calibrating noise to sensitivity in private data analysis, in *Theory of Cryptography Conference*, S. Halevi, T. Rabin, Eds. (Springer, 2006), pp. 265–284.

187. R. Hall, S. E. Fienberg, Privacy-preserving record linkage, in *Proceedings of the 2010 International Conference on Privacy in Statistical Databases* (Springer, 2010), pp. 269–283.

188. D. Vatsalan, P. Christen, V. S. Verykios, A taxonomy of privacy-preserving record linkage techniques. *Inform. Systs.* **38**, 946–969 (2013).

189. D. Vatsalan, Z. Sehili, P. Christen, E. Rahm, Privacy-preserving record linkage for big data: Current approaches and research challenges, in *Handbook of Big Data Technologies*, A. Y. Zomaya, S. Sakr, Eds. (Springer International Publishing, 2017), pp. 851–895.

190. W. Jiang, C. Clifton, A secure distributed framework for achieving k-anonymity. *VLDB J.* **15**, 316–333 (2006).

191. N. Mohammed, B. C. Fung, M. Debbabi, Anonymity meets game theory: Secure data integration with malicious participants. *VLDB J.* **20**, 567–588 (2011).

192. N. Mohammed, D. Alhadidi, B. C. Fung, M. Debbabi, Secure two-party differentially private data release for vertically partitioned data. *IEEE Trans. Dependable Secure Comput.* **11**, 59–71 (2014).

193. X. Cheng, P. Tang, S. Su, R. Chen, Z. Wu, B. Zhu , Multi-party high-dimensional data publishing under differential privacy. *IEEE Trans. Knowledge Data Eng.* **32**, 1557–1571 (2020).

194. M. Wilke, E. Rahm, Towards multi-modal entity resolution for product matching, in *Proceedings of the 32nd GI-Workshop on Foundations of Databases* (*Grundlagen von Datenbanken*) (GVDB, 2021).

195. H. Köpcke, E. Rahm, Frameworks for entity matching: A comparison. *Data Knowledge Eng.* **69**, 197–210 (2010).

196. F. Gregg, D. Eder, Dedupe (2015); https://github.com/dedupeio/dedupe [retrieved 29 July 2020].

197. J. de Bruin, recordlinkage 0.14 (2019); https://pypi.org/project/recordlinkage/ [released 1 December 2019; retrieved 29 July 2020].

198. P. Christen, Febrl—An open source data cleaning, deduplication and record linkage system with a graphical user interface, in *Proceedings of the 14th ACM International Conference on Knowledge Discovery and Data Mining* (Association for Computing Machinery, 2008), pp. 1065–1068.

199. Y. Govind, P. Konda, P. Suganthan, P. Martinkus, P. Nagarajan, H. Li, A. Soundararajan, S. Mudgal, J. R. Ballard, Entity matching meets data science: A progress report from the magellan project, in *Proceedings of the 2019 International Conference on Management of Data* (Association for Computing Machinery, 2019), pp. 389–403.

200. P. Konda, S. Das, P. Suganthan G. C., A. H. Doan, A. Ardalan, J.R. Ballard, H. Li, F. Panahi, H. Zhang, J. Naughton, S. Prasad, G. Krishnan, R. Deep, V. Raghavendra, Magellan: Toward building entity matching management systems. *Proc. VLDB Endowment* **9**, 1197–1208 (2016).

201. M. Sariyar, A. Borg, The RecordLinkage package: Detecting errors in data. *R J.* **2**, 61–67 (2010).

202. M. Friedrichs, C. Webster, B. Marsh, J. Dice, S. Lee, fedmatch: Fast, flexible, and user-friendly record linkage methods (2021). R package version 2.0.3.

203. R. Linacre, S. Lindsay, splink: Probabilistic record linkage and deduplication at scale; https://github.com/moj-analytical-services/splink (2021).

204. L. Gagliardelli, G. Simonini, D. Beneventano, S. Bergamaschi, Sparker: Scaling entity resolution in spark, in *EDBT 2019: 22nd International Conference on Extending Database Technology* (PRT, 2019).

205. G. Papadakis, L. Tsekouras, E. Thanos, G. Giannakopoulos, T. Palpanas, M. Koubarakis, The return of JedAI: End-to-end entity resolution for structured and semi-structured data. *Proc. VLDB Endowment* **11**, 1950–1953 (2018).

206. K.-N. Tran, D. Vatsalan, P. Christen, Geco: An online personal data generator and corruptor, in *Proceedings of the 22nd ACM International Conference on Information & Knowledge Management* (Association for Computing Machinery, 2013), pp. 2473–2476.

207. M. Bilenko, R. Mooney, Riddle: Repository of information on duplicate detection, record linkage, and identity uncertainty (2006); www.cs.utexas.edu/users/ml/riddle/ [retrieved 29 July 2020].

208. B. Spahn, “Before the American voter,” thesis, Stanford University (2019).

209. J. Tang, A. C. Fong, B. Wang, J. Zhang, A unified probabilistic framework for name disambiguation in digital library. *IEEE Trans. Knowledge Data Eng.* **24**, 975–987 (2012).

210. V. I. Torvik, N. R. Smalheiser, Author-ity 2009—Pubmed author name disambiguated dataset (2009).

211. J. Martin Montull, Inspire: Managing metadata in a global digital library for high-energy physics, in *Research Conference on Metadata and Semantic Research* (Springer, 2011), pp. 269–274.
